# Functional role of PGAM5 multimeric assemblies and their polymerization into filaments

**DOI:** 10.1038/s41467-019-08393-w

**Published:** 2019-01-31

**Authors:** Karen Ruiz, Tarjani M. Thaker, Christopher Agnew, Lakshmi Miller-Vedam, Raphael Trenker, Clara Herrera, Maria Ingaramo, Daniel Toso, Adam Frost, Natalia Jura

**Affiliations:** 1 Cardiovascular Research Institute, University of California San Francisco, San Francisco, CA 94158 USA; 2 Department of Biochemistry and Biophysics, University of California San Francisco, San Francisco, CA 94158 USA; 3 Tetrad Graduate Program, University of California San Francisco, San Francisco, CA 94158 USA; 4 Calico Life Sciences, South San Francisco, CA 94080 USA; 5 California Institute for Quantitative Biosciences, University of California Berkeley, Berkeley, CA 94720 USA; 6 Chan Zuckerberg Biohub, San Francisco, CA 94158 USA; 7 Department of Cellular and Molecular Pharmacology, University of California San Francisco, San Francisco, CA 94158 USA

## Abstract

PGAM5 is a mitochondrial protein phosphatase whose genetic ablation in mice results in mitochondria-related disorders, including neurodegeneration. Functions of PGAM5 include regulation of mitophagy, cell death, metabolism and aging. However, mechanisms regulating PGAM5 activation and signaling are poorly understood. Using electron cryo-microscopy, we show that PGAM5 forms dodecamers in solution. We also present a crystal structure of PGAM5 that reveals the determinants of dodecamer formation. Furthermore, we observe PGAM5 dodecamer assembly into filaments both in vitro and in cells. We find that PGAM5 oligomerization into a dodecamer is not only essential for catalytic activation, but this form also plays a structural role on mitochondrial membranes, which is independent of phosphatase activity. Together, these findings suggest that modulation of the oligomerization of PGAM5 may be a regulatory switch of potential therapeutic interest.

## Abstract (web summary)

PGAM5 is a mitochondrial protein phosphatase whose functions include regulation of mitophagy and cell death. Here, the authors use x-ray crystallography and EM to show that PGAM5 forms dodecameric rings and filaments in solution, and find that PGAM5 rings are essential for catalysis and for a structural effect PGAM5 has on mitochondrial membranes, independently of catalytic activity.

## Introduction

**Fig. 1:**
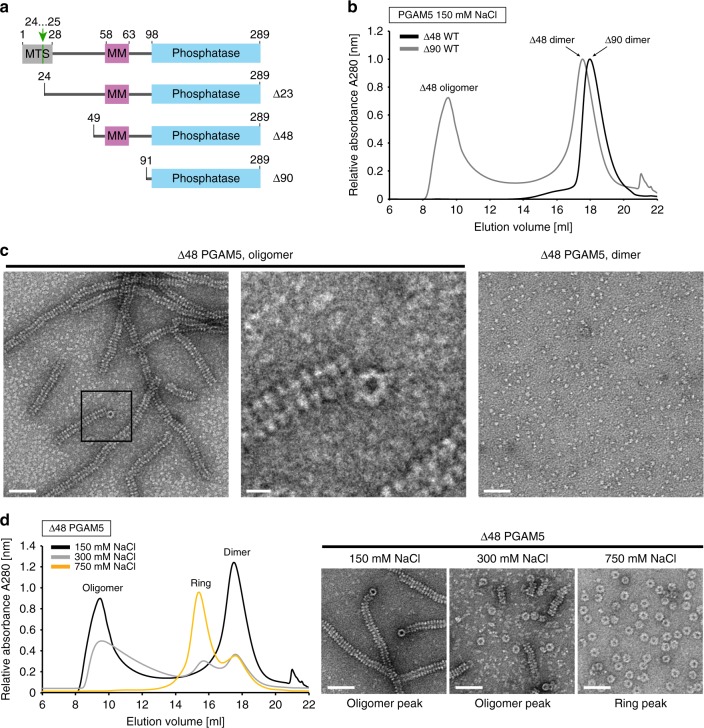
∆48 PGAM5 forms tubular filaments in solution composed of ring-like structures. **a** Domain architecture of constructs used in this study. Full-length PGAM5 is comprised of a single transmembrane helix containing a mitochondrial targeting sequence (MTS), a linker domain including the regulatory multimerization motif (MM), and a C-terminal PGAM phosphatase domain. The native cleavage site between residues 24 and 25, cleaved by PARL^6^, is marked with a green arrow. **b** Elution profiles for ∆48 and ∆90 PGAM5 constructs purified by size exclusion chromatography (SEC) using a Superose 6 column (GE Healthcare) in SEC buffer containing  20 mM Tris-Cl pH 8.0, 500 µM TCEP, and 150 mM NaCl. The corresponding oligomeric states of each peak observed in the chromatograms are indicated. **c** Representative EM micrographs of negatively stained protein samples taken from fractions corresponding to the two distinct peaks observed in the ∆48 PGAM5 purification. **d** SEC profiles for ∆48 PGAM5 in SEC buffer and NaCl at a final concentration of 150, 300, or 750 mM. EM micrographs of negatively stained samples of ∆48 PGAM5 taken directly from the primary peak obtained during purification are shown, highlighting the decomposition of filaments into rings at increasing salt concentrations. Scale bars in **c** and **d** correspond to 50 nm, except for the inset in **c** in which the scale bar corresponds to 10 nm

Phosphoglycerate mutase family member 5 (PGAM5) is an atypical protein phosphatase implicated in a number of functions within mitochondria, including organelle homeostasis, mitophagy, and cell death. As a member of the PGAM histidine phosphatase superfamily, PGAM5 has the conserved PGAM domain^1^. However, unlike most PGAM enzymes, which are phosphotransferases or phosphohydrolases of small metabolites, PGAM5 dephosphorylates protein substrates^2^, targeting phosphorylated serine, threonine, and histidine residues^3,4^. PGAM5 contains an N-terminal mitochondrial targeting sequence (MTS), which also serves as a membrane anchor. Experimental evidence supports PGAM5 localization to the outer mitochondrial membrane (OMM), where its phosphatase domain is accessible from the cytosol^5^ and to the inner mitochondrial membrane (IMM)^6,7^. In response to loss of mitochondrial membrane potential (Δ*Ψ*_m_)^6^ or apoptosis^8,9^, PGAM5 undergoes cleavage by two IMM-resident proteases, presenilin-associated rhomboid-like protein (PARL)^6^, and the metalloprotease OMA1^10^, generating a fragment of PGAM5 missing the MTS (Fig. 1a) that can be partially released into the cytosol^6,8,9,11^. Thus, the localization of PGAM5 seems dynamic and stress responsive.

PGAM5 has been linked to mitochondrial homeostasis and cell death through the regulation of mitochondrial dynamics, respiration and mitophagic quality control. Interaction partners of PGAM5 include KEAP1, an adaptor protein for the Cul3-dependent E3 ubiquitin ligase complex, and Bcl-XL, an anti-apoptotic protein^12^. By inhibiting KEAP1 activity^5^, PGAM5 promotes retrograde transport of mitochondria along microtubules by the GTPase Miro2^13^. PGAM5 also regulates levels of PTEN-induced kinase 1 (PINK1), a guardian of mitochondrial quality control^7,14^, which upon mitochondrial damage accumulates on the cytosolic surface of mitochondria to recruit Parkin and initiate mitophagy^15–18^. In mice, PGAM5 stabilizes PINK1 and promotes mitophagy to protect against neuronal degeneration^7^, whereas in flies PGAM5 negatively regulates PINK1^14^. In response to hypoxia or loss of mitochondrial membrane potential, PGAM5 binds and dephosphorylates FUNDC1, promoting recruitment of autophagosomes to mitochondria^19^, consistent with PGAM5’s role in promotion of mitophagy. Notably, PGAM5 knockout in mice leads to a more severe Parkinson’s disease-like phenotype than observed for PINK1 knockout, suggesting that PGAM5 has multiple functions that support dopaminergic neuron survival^7,20–23^.

The role of PGAM5 in cell death is poorly understood^9,24,25^. In necroptosis, initiated by tumor necrosis factor alpha (TNFα), PGAM5 may be a part of a complex with the kinases RIP1 and RIP3 that promotes cell death through dephosphorylation of the mitochondrial fission factor DRP1^24^. However, the PGAM5/DRP1 axis is dispensable for TNFα-induced necroptosis in many cells and conditions^26–29^, except for ConA-induced hepatic necrosis^30^. Other studies link PGAM5 to apoptosis through sequestration of a caspase inhibitor, XIAP E3 ubiquitin ligase^9^. During apoptosis, PGAM5 also contributes to mitochondrial recruitment of Bax and to DRP1 dephosphorylation, possibly by engaging them in a heteromeric complex^31^. Most recently, PGAM5 was shown to protect cells from necroptosis through its positive effect on PINK1-dependent mitophagy^32^.

Biochemical and structural studies elude to an elaborate mechanism by which many functions of PGAM5 might be controlled. Between the MTS and phosphatase domain, PGAM5 contains a linker domain (Fig. 1a) with a centrally located “multimerization motif” that potentiates PGAM5 activity^33^. The multimerization motif (MM) also promotes higher-order oligomerization of PGAM5^33^. A crystal structure of PGAM5 containing the linker and phosphatase domains showed that the MM stabilizes a catalytically active conformation of the enzyme^34^. The structure also revealed a dodecameric, ring-shaped organization of the phosphatase domain in the crystal lattice^34^, of unknown physiological relevance.

Here, we provide evidence that oligomerization is essential for PGAM5 catalysis and present a new crystal structure of the PGAM5 ring demonstrating the sufficiency of the MM for PGAM5 dodecamer formation. We also discover that PGAM5 can adopt different oligomeric states in solution, including filamentous structures composed of stacked dodecameric rings that can form both in vitro and in cells. Lastly, we find that PGAM5 oligomerization exerts a structural effect on mitochondrial membranes that is independent of its phosphatase activity. Taken together, we propose that the functions of this atypical phosphatase are regulated through both catalytic and noncatalytic mechanisms.

## Results

### Linker domain induces filamentation of the PGAM5 phosphatase

To understand the mechanism of oligomerization-dependent activation of PGAM5, we purified a truncated form of PGAM5 (Δ48 PGAM5), previously characterized as a stable and fully active construct, which includes the MM (residues 58–63) (Fig. 1a)^33^. We focused on the form of PGAM5, known as PGAM5L, that has an intact PGAM domain formed by residues 98–289. Δ48 PGAM5 resolved as two distinct peaks during size exclusion chromatography (SEC) (Fig. 1b). The later peak was consistent with the profile expected for a dimeric complex of PGAM5, similar to the elution observed for the isolated phosphatase domain (Δ90 PGAM5)^33^ (Fig. 1b). The earlier peak, however, was consistent with a larger oligomer that has been observed in biochemical preparations of a comparable construct, Δ54 PGAM5^33,34^.

Analysis of the chromatogram peaks using negative-stain electron microscopy (EM) revealed the presence of spontaneously formed filaments of several hundred nanometers in the earlier peak. These filaments were not observed in purified samples of PGAM5 dimers (Δ48 later peak or Δ90 PGAM5) (Fig. 1c). Filaments were comprised of a repeating unit with an occasional shift in register along the length of the filament. In addition, singular ring-like particles frequently decorated the ends of the filaments in an orientation perpendicular to the filament axis. We hypothesized that the filaments are comprised of individual rings, with the lumen of the ring forming the interior of the filament. The lack of isolated rings within either sample suggests that PGAM5 oligomerization into filaments is a cooperative process.

The presence of rings and their potential role as the building blocks of PGAM5 filaments was intriguing, especially in light of a recent crystal structure of ∆54 PGAM5, which revealed a ring-like oligomer through lattice interactions between 12 copies of PGAM5 (PDB: 5MUF)^34^. ∆48 PGAM5 filaments could be dissociated into rings by increasing the ionic strength (Fig. 1d). With high concentrations of NaCl (>500 mM NaCl), we could enrich for discrete PGAM5 rings, enabling structural analyses on the ring-like ∆48 PGAM5 state in solution.

### PGAM5 rings in solution and in a new crystal structure

**Fig. 2:**
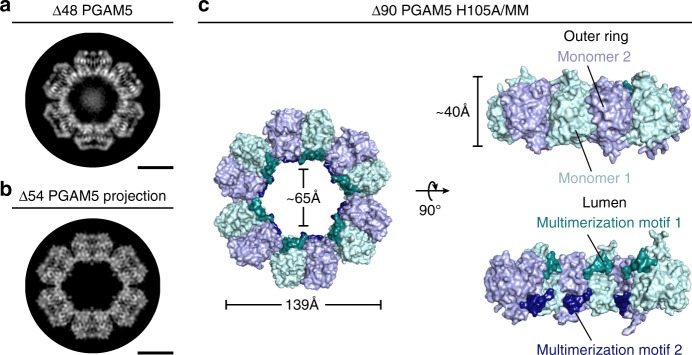
Architecture of the PGAM5 dodecameric ring in solution and in a new crystal structure. **a** 2D class average of the predominant view of the ring assembly observed in cryo-EM micrographs of ∆48 PGAM5 reveals a dodecameric topology highly similar in architecture to **b**. **b** 2D projection image of the crystallographic ring observed in the crystal lattice of ∆54 PGAM5 (PDB: 5MUF) limited to 4.5 Å resolution. **c** Architecture and subunit arrangement of the dodecameric assembly observed in the crystal structure of the ∆90 PGAM5 H105A phosphatase domain crystallized in the presence of a peptide containing the multimerization motif (MM)

We assessed the molecular structure of ∆48 PGAM5 rings in solution by cryo-EM. Our data revealed a mixture of PGAM5 ring particles with dimensions consistent with the formation of single rings, a doublet of rings, and less frequently, partial doublets (Supplementary Figure 1). The particles adopted a preferred orientation in the vitreous layer that limited the number of side views necessary for complete 3D reconstruction. Thus, we performed 2D classification of the top view of the ∆48 PGAM5 ring, visualized along the central symmetry axis, revealing features of PGAM5 at a resolution sufficient to analyze its organization (Fig. 2a). The solution structure of ∆48 PGAM5 adopted C6 symmetry in which six phosphatase domain dimers form a ring. Comparison of the best 2D class of the ∆48 PGAM5 ring to a projection image of the crystallographic ring observed in the structure of ∆54 PGAM5^34^ (filtered to the same 4.5 Å resolution as the 2D average) showed similar ring architectures (Fig. 2b). Despite slightly different dimensions, stemming from the crystallographic symmetry of the ∆54 PGAM5 structure (Supplementary Figure 2a–d), the overall similarity in ring architecture verified that the dodecameric assembly of PGAM5 observed in the crystalline lattice of ∆54 PGAM5 also occurs in solution.

In the Δ54 PGAM5 crystal structure, the linker domain is mostly disordered, except for a region corresponding to the MM^34^. Similarly, we could not clearly identify the linker domain in 2D classes of the Δ48 PGAM5 ring (Fig. 2a). These observations suggested that the interactions supported by the MM might be sufficient to drive oligomerization and activation of PGAM5. This model is supported by a crystal structure we determined for a shorter fragment of a catalytically dead PGAM5 phosphatase domain (Δ90 PGAM5 H105A), in complex with a peptide encompassing the MM (linker domain residues 54–67) (Table 1, Fig. 2c). The asymmetric unit of the unit cell in our 2.6 Å resolution structure of ∆90 PGAM5 H105A/MM revealed organization of the phosphatase domain into a dodecameric ring, with the MM peptide positioned in the lumen of the ring at the base of each phosphatase domain (Fig. 2c). Hence, our structure demonstrates that the MM is sufficient to induce the formation of a dodecameric PGAM5 ring, even when presented *in trans*.

**Table 1:** Data collection and refinement statistics

	∆90 PGAM5 H105A/MM	∆90 PGAM5 H105A
*Data collection*		
PDB ID	6CNL	6CNI
Space group	*P* 2_1_ 2_1_ 2_1_	*P* 2_1_ 2_1_ 2_1_
Cell dimensions		
* a*, *b*, *c* (Å)	49.4, 242.5, 272.5	71.0, 72.0, 81.9
*α*, *β*, *γ* (°)	90, 90, 90	90, 90, 90
Resolution (Å)	48.6 – 2.6 (2.69 – 2.6)	41.0–1.7 (1.76 – 1.7)
* R*_sym_ or *R*_merge_	19.8 (105.8)	7.5 (76.0)
* I*/*σI*	7.2 (1.4)	21.8 (2.3)
Completeness (%)	99.8 (99.9)	99.7 (97.5)
Redundancy	5.0 (5.1)	12.1 (7.8)
*Refinement*		
Resolution (Å)	48.6–2.6 (2.69–2.6)	41.0–1.7 (1.76–1.7)
No. of reflections	509,660 (51,383)	564,588 (35,185)
*R*_work_/*R*_free_, %	22.3/26.4 (30.9/33.6)	17.1/19.7 (25.9/31.3)
No. of atoms		
Protein	18,643	2893
Ligand/ion	31	16
Water	199	308
*B*-factors		
Protein	35.5	26.2
Ligand/ion	30.5	25.8
Water	31.8	37.4
R.M.S. deviations		
Bond lengths (Å)	0.004	0.007
Bond angles (°)	0.8	1.1
Ramachandran plot, %		
Favored	97.8	99.4
Additional allowed	2.2	0.00

Values in parentheses are for highest-resolution shell

### The active conformation is independent of phosphate binding

**Fig. 3:**
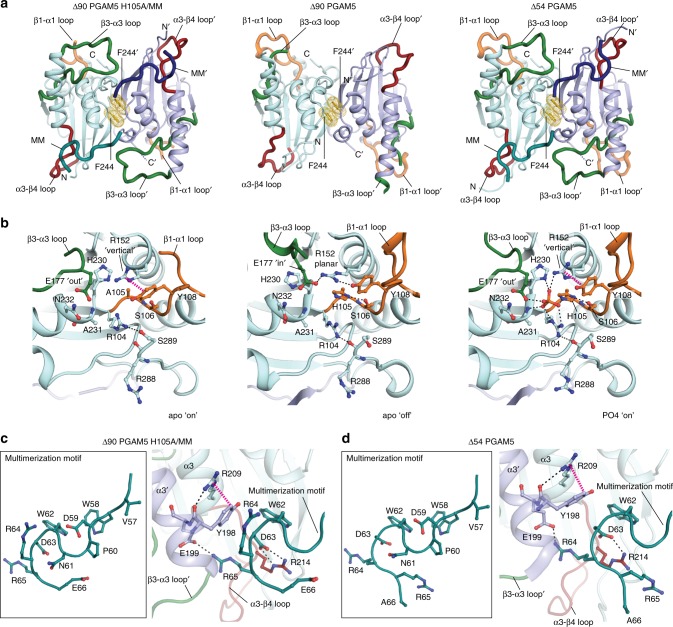
Key features of PGAM5 activation in the crystal structure of ∆90 PGAM5 H015A/MM. **a** The structures of ∆90 PGAM5 H105A with the multimerization motif (MM) added in *trans* (∆90 PGAM5 H105A/MM) (left panel) compared to the structures of ∆90 PGAM5 (PDB: 3MXO) (middle panel) and ∆54 PGAM5 with the MM present in *cis* (PDB: 5MUF) (right panel). Monomers 1 and 2 are colored cyan and light blue, respectively, in all structures with their corresponding MM regions colored in teal (monomer 1 MM) and dark blue (monomer 2 MM) where present. The β1–α1 loop is indicated in orange, the β3**–**α3 loop is indicated in green, the α3–β4 loop is indicated in red. The F244 residues in the central axis forming the dimer interface are indicated in yellow. **b** Detailed view of the catalytic core in ∆90 PGAM5 H105A/MM (left), ∆90 PGAM5 (middle), and ∆54 PGAM5 (right) highlighting interactions between active-site residues and the phosphate ion (PO_4_). **c**, **d** Comparison of the MM architecture (left panel) and the differences in interactions with the phosphatase domain (right panel) for **c** ∆90 PGAM5 H105A/MM and **d** ∆54 PGAM5

The dodecameric ring in the crystal structure of ∆90 PGAM5 H105A/MM has a central six-fold symmetry axis that better matches the C6 symmetry of the 2D class of the ∆48 PGAM5 ring than the C2 symmetry of the ∆54 PGAM5 crystallographic dodecamer (Fig. 2b). The differences between the two ring geometries stem from a slight rotation of the PGAM5 phosphatase monomers at the α3 helical interface; whereas the dimer interface-flanking residues F244 and burying approximately 1050.5 Å^2^ through hydrophobic interactions is the same in both structures (Supplementary Figure 2e). Despite the differences, the ordered β3–α3 catalytic loop (residues 172–193) indicated that the phosphatase domain in both structures is in the active conformation (Fig. 3a). A notable difference between the ∆54 PGAM5 structure and the ∆90 PGAM5 H105A/MM structure occurred in the β1–α1 loop (residues 105–121). In the Δ54 PGAM5 structure, this loop is ordered, whereas it is missing in the ∆90 PGAM5 H105A/MM dodecamer. This difference could be due to the engagement of the β1–α1 loop in crystal contacts in the Δ54 PGAM5 structure, but not in the ∆90 PGAM5 H105A/MM structure.

Mutation of the catalytic histidine (H105) to alanine resulted in active-site arrangements in our ring structure of ∆90 PGAM5 resembling an active state described as the PO4 “on” conformation in the ∆54 PGAM5 structure^34^ (Fig. 3b, left and right panels). In this on state, the H230 residue is positioned inward relative to its position in the structure of the inactive phosphatase domain alone (∆90 PGAM5 (PDB:3MXO); Fig. 3b, center panel). In the ∆54 PGAM5 structure residue R152 adopts a vertical, rather than the planar position observed in the ∆90 PGAM5 dimer structure^34^, forming cation-π–stacking interactions with Y108, and together with H230 and H105, coordinates an active-site phosphate (Fig. 3b, right panel). In the ∆90 PGAM5 H105A/MM structure, residues R152, Y108, and H230 adopt similar orientations, but in the absence of phosphate (Fig. 3b, left panel). The on state observed for ∆90 PGAM5 H105A/MM in the absence of bound phosphate underscores the importance of ring assembly for stabilizing the active architecture of the catalytic site.

Chaikuad et al.^34^ suggested that capping of the active site by the β3–α3 loop positions active-site residues in the catalytically competent state. However, a crystal structure of Δ90 PGAM5 H105A without the MM that we determined suggests otherwise (Table 1, Supplementary Figure 3). In this structure, PGAM5 phosphatase formed a dimer analogous to the one previously observed in the structure of Δ90 PGAM5 wild type (PDB:3MXO). Although the crystal packing is identical in the two Δ90 PGAM5 structures, the active-site residues of Δ90 PGAM5 H105A adopt catalytically competent conformations (PO4 “on”) in the presence of a phosphate ion (Supplementary Figure 3b). The main difference between this dimer conformation and the apo “on” state of the dodecameric ∆90 PGAM5 H105A/MM structure is a disordered β3–α3 loop in Δ90 PGAM5 H105A. Thus, in the absence of discrete phosphatase domain interactions with the β3–α3 loop, the active architecture can be achieved, at least structurally, as long as a phosphate ion is coordinated. Altogether, these structures point to a remarkable plasticity of the PGAM5 catalytic center, which indicates that multiple structural elements coordinate for catalysis.

We also observed a notable difference in the conformation of the C-terminal region of the MM between our ∆90 PGAM5 H105A/MM structure and the previously determined ∆54 PGAM5 structure^34^. Although an identical length of the linker is resolved in both, the improved resolution of the ∆90 PGAM5 H105A/MM structure and overall quality of the electron density map in the linker region revealed a register shift in which the positions of R64 and R65 in the ∆54 PGAM5 structure are occupied by R65 and E66 in the ∆90 PGAM5 H105A/MM structure, respectively (Fig. 3c, d; Supplementary Figures 2e, 4). In ∆90 PGAM5 H105A/MM, R64 is positioned directly in the center of a dimerization interface mediated by the α3 helices (Fig. 3c, right panel), expanding the network of interactions in this region. The guanidinium group of R64 in monomer 1 is also involved in a previously unobserved cation π-stacking interaction in *trans* with the phenyl ring of Y198 of the adjacent monomer. Residue E199, previously described as hydrogen bonded to R64 of the adjacent monomer, now interacts with R65. In both structures, R214 hydrogen bonds in *cis* with D63, and R209 with the backbone carbonyl of Y198 in *trans* (Fig. 3c, d).

### Catalytic activation of PGAM5 requires dodecamer formation

**Fig. 4:**
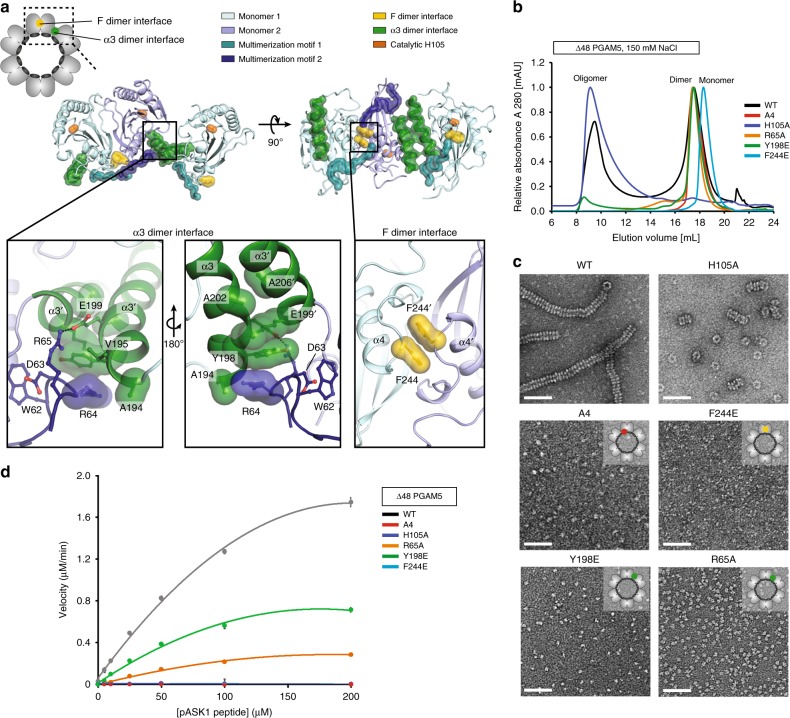
PGAM5 multimerization is required for catalysis. **a** Overview of interactions contributing to the stability of the dodecamer assembly in the structure of ∆90 PGAM5 H105A/MM. **b** SEC elution profiles of wild type (WT) and interface-disrupting mutants (R65A, H105A, Y198E, F244E, and A4) of ∆48 PGAM5 in 150 mM NaCl-containing buffer, highlighting the effect of the MM (R65A, A4), and dimer interface (F244E, Y198E) mutants on the oligomer/dimer equilibria observed for wild-type ∆48 PGAM5. **c** EM micrographs of negatively stained samples of interface mutants of PGAM5 corresponding to the oligomer peaks for WT and H105A, and dimer/monomer peaks for all the other mutants as shown in **b**. The insets in top right corners illustrate the position of the respective mutations within the PGAM5 dodecamer cartoon. Scale bars correspond to 50 nm. **d** Phosphatase activity of the WT and mutant variants of ∆48 PGAM5 measured for 20 nM enzyme and varying concentrations of a phosphorylated ASK1 substrate peptide. Data are represented as mean ± S.E.M. The kinetic parameters *K*_m_, *k*_cat_, and *k*_cat_/*K*_m_ were determined using GraphPad Prism and are summarized in Table 2

We further examined the role of dodecamer formation in the regulation of PGAM5 activity. In PGAM5 ring structures, each MM peptide interacts with three phosphatase domain monomers. An interaction with the central phosphatase subunit involves binding of the WDxxWD sequence of the MM. Two additional minor interactions engage N- and C-terminal ends of the MM with two distinct dimer interfaces formed by the neighboring phosphatase domains (Fig. 4a). We defined the first minor interaction as the F dimer interface because of the presence of a pair of centrally located F244 residues supporting the dimerization interface. We defined the second interaction as the α3 dimer interface, because it involves a phosphatase domain dimer that engages the α3 helices. We introduced the F244E mutation to disrupt the F dimer interface and Y198E or R65A mutations to disrupt the α3 dimer interface (Fig. 4b). We also generated an A4 mutant of PGAM5 in which residues WDxxWD of the MM are replaced by the AAxxAA sequence, which inactivates PGAM5^33^.

All four mutations affected Δ48 PGAM5 oligomerization (Fig. 4b, c). The F244E mutation had the most dramatic effect, shifting the entire population of Δ48 PGAM5 to a monomeric state. Likewise, the A4 mutant did not engage in higher-order oligomerization and, instead, preferentially formed dimers. Like the A4 mutant, both the Y198E and R65A mutants were predominantly or completely dimers. Because none of the A4, Y198E, or R65A mutations altered the F dimer interface, we predicted that their dimeric state is likely stabilized by the F244-centered interface (Fig. 4a). These findings suggested that, although both the F dimer and α3 dimer interfaces are important for ring formation, only the F244-mediated dimerization occurs in the absence of dodecamer formation.

We determined the catalytic activity of the PGAM5 interface mutants, including the H105A and A4 mutants as negative controls, by measuring the rate of PGAM5-mediated dephosphorylation of a phospho-ASK1 substrate peptide (Table 2). The monomerizing F244E mutation resulted in complete loss of phosphatase activity (Fig. 4d). The Y198E and R65A mutations, which dissociated ∆48 PGAM5 ring oligomers without disrupting the F dimer, also impaired phosphatase activity, decreasing catalytic efficiency ~two and ~fivefold, respectively (Fig. 4d, Table 2). The negative effect of every mutation disrupting PGAM5 ring assembly on phosphatase activity indicated that dodecamer formation is necessary to achieve maximal catalytic efficiency. These results also showed that phosphatase activity primarily depends on the interaction between the MM and phosphatase domains through the F dimer interface and that organization of F dimers into a hexameric ring through the α3 dimer interface further potentiates activity.

**Table 2:** Phosphatase activity of PGAM5 and its oligomerization-deficient mutants

Protein	*K*_m_ (µM)	*K*_cat_ (min^−1^)	*K*_cat_/*K*_m_ (min^−1^ M^−1^)
∆48 WT	115.2 ± 5.0	137.20 ± 2.98	1.19 (±0.06) × 10^6^
∆48 H105A	ND	ND	ND
∆48 R288E	174.70 ± 26.99	143.30 ± 12.73	0.82 (± 0.15) × 10^6^
∆48 F244E	ND	ND	ND
∆48 A4	ND	ND	ND
∆48 R65A	119.9 ± 15.7	23.17 ± 1.55	0.19 (±0.03) × 10^6^
∆48 Y198E	86.17 ± 6.19	51.65 ± 1.68	0.60 (±0.05) × 10^6^

Dephosphorylation of the ASK1 phosphopeptide by WT and mutant variants of ∆48 PGAM5 was measured at 20 nM of enzyme concentration and varying concentrations of ASK1 substrate. The kinetic parameters *K*_m_, *k*_cat_, and *k*_cat_/*K*_m_ were determined using GraphPad Prism software. Kinetic data for PGAM5 variants without measurable phosphatase activity are denoted ND (not determined). Data are represented as mean ± S.E.M.

### Filament formation does not enhance PGAM5 activity

**Fig. 5:**
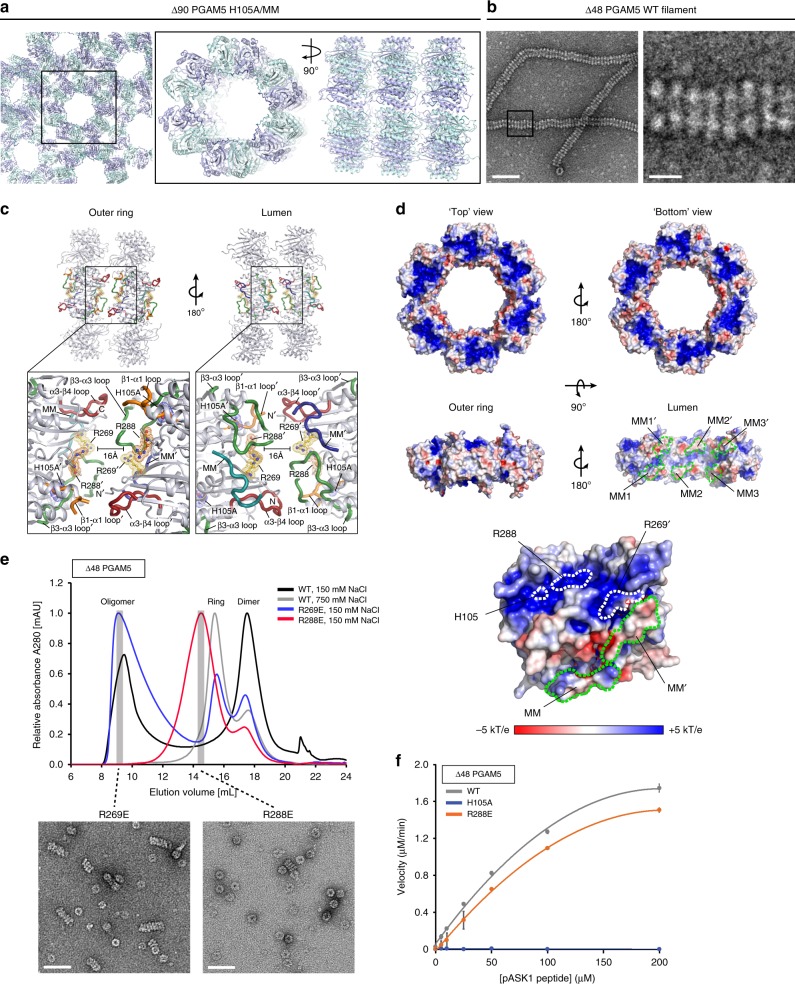
Role of PGAM5 filaments in catalysis. **a**, **b** Overview of the **a** crystal packing in the ∆90 PGAM5 H105A/MM structure (left panel) highlighting a network of stacked rings (right panel), which resembles the organization of **b** filaments observed for ∆48 PGAM5 in solution by negative-stain EM. **c** Overview of the ring stacking interface highlighting the positions of residues at the surface of the substrate-binding cleft relative to structural elements contributing to peripheral interactions between two adjacent dodecamers within the crystal lattice of the ∆90 PGAM5 H105A/MM structure. **d** Electrostatic surface potential calculated for the ∆90 PGAM5 H105A/MM dodecamer using APBS^71^. The positions of the MM outlined in green are shown relative to the positions of basic residues R269E and R288E within the positively charged substrate-binding cleft as well as the catalytic residue, H105, in the active site. Coloring corresponds to the electronegativity of the surface potential as defined in the scale bar, with more negatively charged surfaces colored red and positively charged surfaces colored blue. **e** Upper panel: size exclusion chromatograms of the WT ∆48 PGAM5 purified in buffer containing low (150 mM NaCl) or high (750 mM NaCl) salt concentrations compared to the results obtained for the R269E and R288E variants. Lower panel: EM micrographs of the negatively stained samples of mutant ∆48 PGAM5 taken from the corresponding primary peak of the purification. **f** Phosphatase activity of the wild type (WT), catalytically inactive H105A, and the stacking-impaired R288E variants of ∆48 PGAM5 measured for 20 nM enzyme and varying concentrations of a phosphorylated ASK1 substrate peptide. Data are represented as mean ± S.E.M. The kinetic parameters *K*_m_, *k*_cat_, and *k*_cat_/*K*_m_ were determined using GraphPad Prism and are summarized in Table 2. Scale bars in **b** and **e** correspond to 50 nm, except for the inset in **b** in which the scale bar corresponds to 10 nm

In solutions of physiological ionic strength, ∆48 PGAM5 existed predominantly as filaments composed of repeating dodecameric rings (Fig. 1c). Although such structures are not present in the crystal lattice of ∆54 PGAM5^34^, the unit cell of our ∆90 PGAM5 H105A/MM structure reveals rings stacked to form tubular filaments (Fig. 5a). Within the filament, each successive ring is rotated ~15° around the 6-fold symmetry axis relative to the neighboring ring. This architecture resembles the filament organization of ∆48 PGAM5 examined by negative-stain EM (Fig. 5b). In the crystal structure, the lattice interactions that mediate ring stacking lack an extensive buried surface area and rely on sparse and nonsymmetrically distributed hydrogen bond interactions between residues within loops at the perimeter of the rings (Fig. 5c and Supplementary Figure 5a). This could explain the apparent lack of structural rigidity in the EM images (Fig. 5b). Mutation of the hydrogen-bonding residues did not have a measurable effect on formation of the ∆48 PGAM5 filaments in solution (Supplementary Figure 5b), suggesting that stabilization of filament formation likely occurs through forces independent of loop interactions observed in the crystal structure.

The twofold rotational symmetry relating individual monomers within each phosphatase dimer results in identical electrostatic surface potentials on both sides of the dodecameric assembly (Fig. 5d). Basic residues comprise a large positively charged patch centered on the catalytic clefts of each dimer surrounded by somewhat negatively charged protruding loops. The structural and electrostatic symmetry suggests that filament formation lacks directionality and can occur from either face of the ring. These characteristics, together with the disruptive effect of high-ionic strength on PGAM5 filaments (Fig. 1d), implicate complementary electrostatic forces in stabilizing the formation of PGAM5 filaments in vitro. To test this, we designed two charge-reversing mutations within the basic cleft, R269E and R288E, and examined their influence on PGAM5 filamentation. In buffers with physiological salt concentrations, each mutation reduced PGAM5 filament length and formation, with the R288E mutation having the greater effect (Fig. 5e). With the R288E mutant, we then investigated the role filaments play in regulating phosphatase activity. The phosphatase activities were similar for both wild type and R288E ∆48 PGAM5, suggesting that filament formation does not contribute substantially to the catalytic efficiency of PGAM5 phosphatase activity (Fig. 5f, Table 2).

### PGAM5 forms large oligomers in cells upon cleavage from IMM

**Fig. 6:**
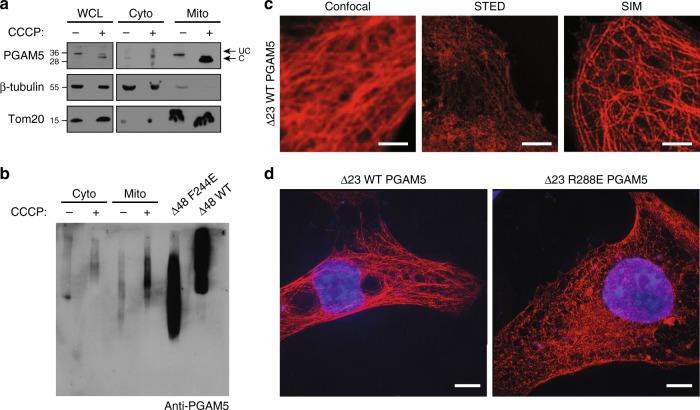
Cleaved PGAM5 forms oligomers in cells. **a** SDS-PAGE analysis of endogenous PGAM5 in HEK293T whole cell lysate (WCL), mitochondrial (mito), and cytoplasmic (cyto) fractions 4 h post-CCCP treatment. Uncleaved (UC) and cleaved (C) PGAM5 bands are marked by arrows. **b** BN-PAGE analysis of endogenous PGAM5 4 h post-CCCP treatment in mitochondrial (mito) and cytoplasmic (cyto) fractions. Fractionation samples were normalized based on the total protein concentrations determined for the WCL samples. As a reference for migration behavior of PGAM5 oligomers, recombinant ∆48 WT (∆48 WT) and the oligomerization-deficient mutant ∆48 F244E mutant (∆48 F244E) PGAM5 were run alongside cellular fractionation samples on a 4–16% native-PAGE acrylamide gel (Invitrogen). Following electrophoresis, gels were immunoblotted with anti-PGAM5 antibody. **c** Comparison of myc-tagged ∆23 PGAM5 filaments in transiently transfected COS7 cells stained with anti-myc antibody using three different imaging methods: confocal microscopy (left panel), stimulated emission depletion (STED) microscopy (middle panel), and structured illumination microscopy (SIM) (right panel). All scale bars correspond to 5 µm. **d** SIM images of myc-tagged ∆23 PGAM5 filaments in COS7 cells transiently expressing wild type (WT) or the R288E PGAM5 (red). Nuclei were stained using DAPI (blue). Both scale bars in **d** correspond to 10 µm

We investigated whether PGAM5 also organizes into filaments in living cells. Our biochemical and structural analysis of the filaments focused on PGAM5 without its membrane-anchoring helix. This construct approximates the state of PGAM5 generated by PARL or OMA1 cleavage in response to the loss of mitochondrial membrane potential upon treatment with CCCP, a small molecule inhibitor of oxidative phosphorylation. The resulting cleaved PGAM5 is mainly detected in mitochondria, with only a fraction detectable in the cytosol^6,8,9,11^. We explored the subcellular localization and oligomerization of endogenous PGAM5, using CCCP to induce cleavage of PGAM5 in HEK293T cells and subcellular fractionation to distinguish between the cytoplasmic and mitochondrial pools of PGAM5. In CCCP-treated cells, cleaved PGAM5 predominantly partitioned to mitochondria (Fig. 6a). To assess the oligomeric state of mitochondrial PGAM5 before and after cleavage, we used blue native electrophoresis (BN-PAGE). We confirmed that BN-PAGE could differentiate different sizes of PGAM5 oligomers by comparing the migration of recombinant wild type ∆48 PGAM5 and the oligomerization-deficient mutant F244E (Fig. 6b). By comparing the migration of endogenous PGAM5 in the cytoplasmic (cyto) and mitochondrial (mito) fractions of untreated or CCCP-treated cells, we observed markedly higher molecular weight species for the cleaved pool of mitochondrial PGAM5 compared to the uncleaved population, approximating the behavior of the recombinant wild-type ∆48 PGAM5 filaments (Fig. 6b). We observed a similar pattern in the cytosolic fraction after CCCP treatment, although the cytosolic pool represented only a small fraction of the total PGAM5 detected.

To visualize PGAM5 filaments in cells, we focused on cytosolic cleaved PGAM5 due to resolution limits preventing the distinction between membrane-tethered and cleaved PGAM5 molecules in the mitochondrial intermembrane compartment. To increase the pool of the cleaved cytosolic PGAM5, we expressed and imaged myc-tagged ∆23 PGAM5 (residues 24–289), mimicking the cleaved version, in COS7 cells using confocal microscopy. In a small subset of transfected cells, we observed myc-positive wire-like cytosolic structures (Fig. 6c). Using two higher-resolution imaging techniques, stimulated emission depletion microscopy (STED) and structured illumination microscopy (SIM), we visualized PGAM5 filaments throughout the cytoplasm in the subset of cells with this PGAM5 phenotype (Fig. 6c). As a control, we evaluated the PGAM5 phenotype of the filament disrupting mutant, R288E (Fig. 5e). We did not find any cytosolic filaments in cells expressing the R288E mutant of ∆23 PGAM5 (Fig. 6d). All mutations that disrupted dodecamer assembly and consequently formation of PGAM5 filaments in vitro (Fig. 4b, c) also eliminated the detection of filaments in cells (Supplementary Figure 7). Thus, the cellular structures we observed have properties like the PGAM5 filaments observed in vitro.

We noted that the architecture of cleaved PGAM5 filaments formed in cells resemble cytoskeletal structures. To test for a spatial relationship between PGAM5 filaments and cytoskeleton, we assessed PGAM5 filament colocalization with actin fibers or microtubules. Although we did not detect overlap between PGAM5 filaments and actin fibers (Supplementary Figure 8a), PGAM5 filaments colocalized with microtubules (Supplementary Figure 8b). Upon nocodazole treatment to disrupt microtubule polymerization, detection of PGAM5 filaments was lost (Supplementary Figure 9), suggesting a potentially intriguing dependence of PGAM5 filament assembly in cells on PGAM5 association with intact microtubules.

### PGAM5 dodecamers promote mitochondrial clustering

**Fig. 7:**
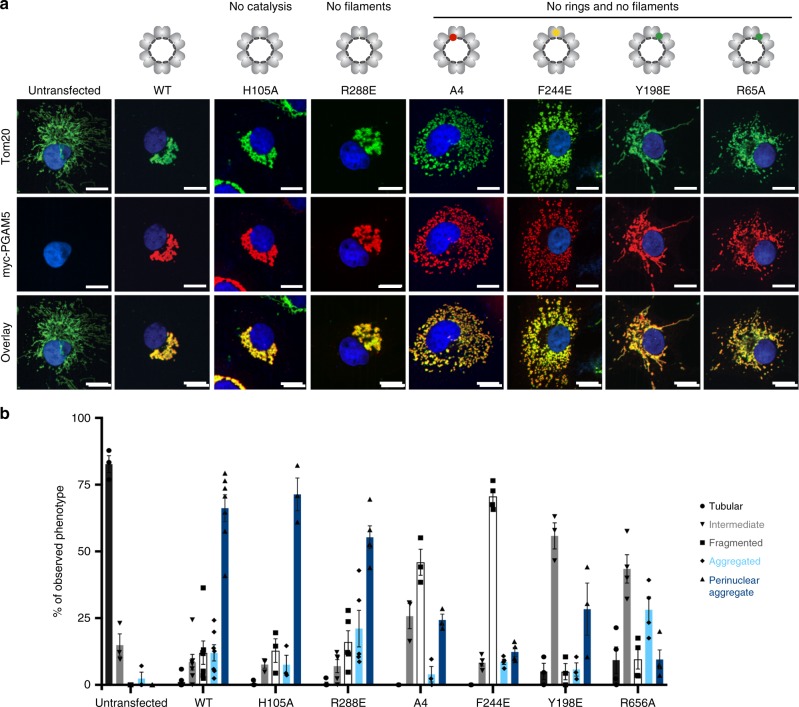
Multimerization of full-length PGAM5 promotes clustered mitochondrial morphology. **a** Representative confocal images of COS7 cells transiently transfected with the wild type (WT) or mutant full-length myc-tagged PGAM5 constructs and immunostained for the OMM marker Tom20 with anti-Tom20 antibody (green), PGAM5 with anti-myc antibody (red), and nucleic-stain DAPI (blue). All scale bars correspond to 15 µm. **b** Quantification of mitochondrial phenotypes observed by confocal microscopy in COS7 cells transiently expressing PGAM5 variants as described in **a**, based on at least three independent experiments per construct. Data are represented as mean ± S.E.M., determined using GraphPad Prism. Untransfected cells: *n* = 122 cells over three experimental replicates. Cells expressing PGAM5 WT: *n* = 296 cells over seven experimental replicates. Cells expressing PGAM5 H105A: *n* = 141 cells over three experimental replicates. Cells expressing PGAM5 R288E: *n* = 212 cells over five experimental replicates. Cells expressing PGAM5 A4: *n* = 144 cells over three experimental replicates. Cells expressing PGAM5 F244E: *n* = 186 cells over four experimental replicates. Cells expressing PGAM5 Y198E: *n* = 108 cells over three experimental replicates. Cells expressing PGAM5 R65A: *n* = 141 cells over four experimental replicates

Under conditions of intact mitochondrial membrane potential, PGAM5 is expected to remain in the uncleaved, membrane-tethered form. To examine the importance of PGAM5 oligomerization in this context, we leveraged the observation that overexpression of full-length PGAM5 induced clustering of mitochondria in the perinuclear region^5^. PGAM5 knockdown has an opposite effect on mitochondria, resulting in increased tubular morphology^26^ and decreased stress-induced mitochondrial clustering^35^. Such effects on mitochondrial behavior stemming from changes in PGAM5 abundance may reflect the role of PGAM5 in maintaining mitochondrial homeostasis^13,26,33^. Using confocal imaging of COS7 cells, we evaluated the colocalization of full-length PGAM5 or PGAM5 mutants and a marker of mitochondrial outer membranes, Tom20, and assessed the effect of full-length PGAM5 expression on mitochondrial morphology (Fig. 7a). Consistent with previous reports^33^, expression of the two catalytically inactive PGAM5 mutants, H105A and A4, had opposing effects on mitochondrial clustering. The membrane-tethered H105A mutant induced mitochondrial clustering, similar to the effect of overexpressing wild type PGAM5; the A4 mutant resulted in fragmentation of the mitochondria (Fig. 7a, b).

The differences in the architecture of recombinant PGAM5 complexes formed by the H105A and A4 mutants could explain their effects on mitochondria. Although both mutations inactivate PGAM5, the H105A mutant can still form higher-order oligomers whereas the A4 mutant prevents multimerization (Fig. 4b, c). Thus, we hypothesized that the assembly of the PGAM5 dodecamer, and perhaps also filamentation, drives changes in mitochondrial morphology. To test this hypothesis, we expressed the F244E mutant, which disrupts the F dimer interface and prevents multimerization, and observed that it failed to induce mitochondria clustering. Instead, the F244E mutant caused mitochondrial fragmentation similar to the A4 mutant (Fig. 7a, b). Expression of both α3 dimer interface mutants (Y198E and R65A), which disrupt ring and filament formation but preserve some phosphatase activity, also failed to induce mitochondrial clustering and resulted in a more tubular phenotype than the A4 or F244E mutants. These results suggested that mitochondrial-clustering phenotype depends on intact dodecamer interfaces of PGAM5, independent from its activity.

To evaluate if assembly of PGAM5 dodecamers into filaments affects mitochondrial clustering, we evaluated the R288E mutation, which disrupted formation of PGAM5 filaments in vitro without affecting the structure of the PGAM5 dodecamer (Fig. 5e). Expression of the R288E mutant induced mitochondrial clustering, suggesting that filament formation is either nonessential for mitochondrial clustering or is precluded when PGAM5 is embedded within mitochondrial membranes (Fig. 7a, b).

### Mitochondrial clustering by PGAM5 is independent of DRP1

DRP1, a cytosolic dynamin-family GTPase that binds to its receptors on the OMM to promote mitochondrial fission^36^, is a substrate of PGAM5^24,33^. We investigated if the clustering effect of PGAM5 overexpression on mitochondrial morphology depends on DRP1 by assessing mitochondrial morphology in *Drp1*^−/−^ mouse embryonic fibroblasts (MEFs) upon PGAM5 overexpression. The smaller size of MEF cells relative to COS7 cells made it difficult to categorize the full spectrum of phenotypes noted for COS7 cells (Fig. 7a, b), thus we limited the phenotypic analysis of MEFs to three categories: (i) tubular/elongated, (ii) fragmented, and (iii) aggregated (Supplementary Figure 10). In untransfected *Drp1*^−/−^ cells, mitochondrial tubules were more connected and elongated^37–39^ than in wild-type MEFs (Supplementary Figure 11a); however, we were still able to identify all three mitochondrial phenotypes (Supplementary Figure 10). The distribution of phenotypes was comparable to that in wild type MEFs, despite differences in the overall appearance of the mitochondrial tubules (Supplementary Figure 11b).

Overexpression of wild-type PGAM5 in wild-type or *Drp1*^−/−^ MEFs resulted in mitochondrial clustering similar to that observed in COS7 cells (Supplementary Figure 11b, c). Expression of F244E PGAM5 in either wild type or DRP1-knockout cells resulted in a shift to the fragmented phenotype of mitochondria (Supplementary Figure 11b, c). Thus, the effects of PGAM5 overexpression on mitochondrial phenotypes are independent of DRP1.

### PGAM5 localizes at contact sites of clustered mitochondria

**Fig. 8:**
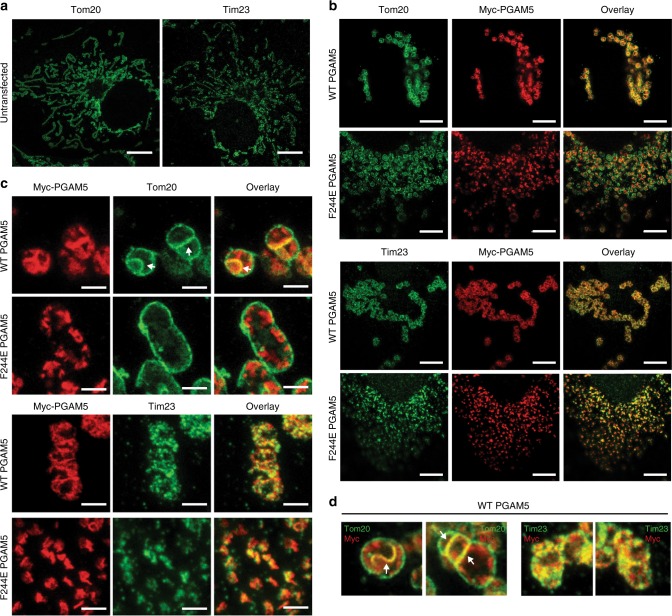
PGAM5 localizes at inner mitochondrial membrane and at the membrane contact sites. **a**–**d** STED images of COS7 cells that were either **a** untransfected or **b**, **c** transiently transfected with wild type (WT) or F244E full-length myc-tagged PGAM5. Tom20 was visualized with anti-Tom20 antibody (green), Tim23 with anti-Tim23 antibody (green), and PGAM5 with anti-myc antibody (red). White arrows indicate contact sites between mitochondrial membranes in which myc-tagged PGAM5 was found colocalized with Tom20. Scale bars correspond to 5 µm in **a**, 5 µm in **b**, and 1 µm in **c**. **d** Upper panels show representative close-up views of PGAM5 localization to the sites of mitochondrial membrane contacts. White arrows indicate sites of PGAM5 (myc, red) overlap with the outer mitochondria membrane marker Tom20 (green)

To characterize the spatial distribution of wild type and F244E PGAM5 at the mitochondria, we used STED to visualize the OMM with Tom20 immunostaining or the IMM with Tim23 immunostaining. In untransfected COS7 cells, mitochondria adopted a phenotype in which tubular Tom20-positive or Tim23-positive structures extended radially from the perinuclear regions (Fig. 8a). In cells transiently transfected with wild-type PGAM5, mitochondrial tubules were replaced by circular mitochondria clustered in the perinuclear region (Fig. 8b). Frequently, single clusters were found stacked next to each, appearing as though these membranes were in direct contact (Fig. 8c). PGAM5 was localized exclusively inside of these distorted mitochondrial structures and detected as puncta that mostly colocalized with Tim23 and not with Tom20 (Fig. 8b, c). We also observed that PGAM5 colocalization with Tom20 only occurred at the sites of contact between individual mitochondrial clusters (Fig. 8b, c).

As with confocal analysis (Fig. 7a), STED imaging showed that F244E PGAM5 expression resulted in a fragmented mitochondrial morphology and more diffuse distribution of mitochondrial vesicles in the cell (Fig. 8b). In contrast to wild type PGAM5, the F244E-positive mitochondria did not engage in any apparent intermitochondrial contacts and PGAM5 in these cells was not colocalized with Tom20. Rather, the F244E mutant was primarily found in Tim23-positive structures (Fig. 8b, c). These data point to a functional importance of PGAM5 multimerization in bridging adjacent mitochondrial membranes to promote interactions that might underlie the clustered phenotype. Our data are also consistent with a localization for PGAM5 at the inner mitochondrial membrane and/or possibly also within the intermembrane space, as evidenced by partitioning of the PGAM5 signal at the inner periphery of the OMM and colocalization with Tim23.

## Discussion

Here, we investigated activation mechanisms of the PGAM5 phosphatase, whose remarkable spectrum of functions in mitochondria parallels the pleiotropic roles of the organelle itself. We discovered that PGAM5 assembles into symmetric rings in solution that polymerize into filaments. We showed that the ring structure, first observed by Chaikuad et al.^34^ for a PGAM5 construct containing its linker and phosphatase domains, also forms when phosphatase domain without these additional contiguous regions is crystallized in the presence of a peptide encompassing only the multimerization motif (MM). Observation of PGAM5 rings in solution indicates that this form is an energetically favored state of the PGAM5 phosphatase.

Our studies demonstrate that the assembly into the dodecameric ring is an essential determinant of efficient catalytic activation of PGAM5. The multimerization step is critical: the interaction between a MM within the linker domain and a pair of phosphatase domains is essential for stabilization of the phosphatase in the active state. This interaction is further stabilized by neighboring subunits in the oligomer. The linker domain of PGAM5 mediates several protein–protein interactions, such as binding of KEAP1 and XIAP ubiquitin ligases^5,9^ and Bcl-XL^12^. It is possible that through such interactions, the linker domain may regulate oligomerization, further tuned by possible post-translational modifications within the linker region. The sufficiency of the presentation of the MM *in trans* could also create a mechanism for propagation of regulatory modifications from one PGAM5 monomer to another, or even between dodecamers, if PGAM5 subunits in the oligomers are capable of exchanging. This could equip PGAM5 with the ability to store information, such as the status of phosphatase domain activation, in a manner analogous to that described for CamKII^40^.

The multimeric organization of PGAM5 might also have important implications for its substrate specificity as a phosphatase. Some PGAM5 substrates form higher-order oligomeric structures, including DRP1, which self-assembles into rings or filaments^36,41^. GTP hydrolysis, an important step in mitochondrial constriction by DRP1, is thought to release DRP1 from its mitochondrial receptors and triggers DRP1 filaments to curl into ring-shaped oligomers, often dodecamers^41^, analogous to the PGAM5 oligomers that we describe here. Although DRP1 associates with the OMM and PGAM5 resides primarily in IMM, we observed that PGAM5 colocalizes with the OMM marker at contact sites between two adjacent mitochondrial vesicles within the mitochondrial clusters promoted by PGAM5 overexpression. These PGAM5-enriched contact sites may represent a type of membrane fusion or fission intermediate involving the formation of local membrane pores^42–47^, which could facilitate interactions between DRP1 and PGAM5.

Mitochondrial morphology and function are tightly linked. The IMM is intricately structured and contains functionally distinct regions, such as inner boundary regions that are close to OMM, cristae invaginations and cristae junctions^48–51^. Formation of inner membrane supercomplexes by IMM proteins, such as adenine nucleotide translocator^52^, ATP synthase^50,53^ and multiprotein Mitofilin complex (MitOS)^54^, is important for structural organization of the functional domains within the IMM. Our data demonstrate that PGAM5 resides primarily in the IMM and acts independently of DRP1 to influence mitochondrial morphology, suggesting that PGAM5 has an intrinsic ability to alter mitochondrial architecture. Furthermore, oligomerization, but not activity, appeared crucial for this ability of PGAM5. Hence, PGAM5 oligomerization within IMM may play a structural role in the maintenance of the functional mitochondrial membrane architecture.

Our finding that cleaved PGAM5 forms filaments composed of dodecameric phosphatase rings both in vitro and in cells adds a new dimension of complexity to PGAM5 signaling. Filaments did not influence the catalytic rate of PGAM5 in vitro nor the ability of full-length PGAM5 to influence mitochondrial morphology in cells. However, cleaved PGAM5, which accumulates with time in the cytosol, sensitizes cells to apoptosis through sequestration of the negative regulator of apoptosis, the ubiquitin ligase XIAP^9^. Other cytosolic effectors of death pathways form filamentous structures that are essential for executing cell death programs, including the caspase-8 associated death complex^55^, the caspase-1 containing inflammasomes^47^ and the RIP1/RIP3 necrosomes^56^. PGAM5 filamentation could ensure highly processive or cooperative dephosphorylation in these processes. We found that cytosolic PGAM5 filaments colocalized with microtubules and only formed when microtubules are intact. This phenomenon may relate to the link between PGAM5 and the stability of the Miro2 GTPase, which involves KEAP1 and the formation of a ternary complex with the Nrf2 transcription factor, to control stress-induced retrograde trafficking of mitochondria^35^. It is also possible that PGAM5 filaments formed upon PGAM5 cleavage reside primarily inside the mitochondria, playing yet unknown role(s).

Although we still know little about the regulation of PGAM5 activity, the enhanced phosphatase activity in oligomerization-competent PGAM5 supports the need for tight regulation of  PGAM5's oligomeric transitions. How binding partners or post-translational modifications regulate PGAM5 catalytic activity and/or oligomerization is a topic of future studies. An exciting possibility is the pharmacological regulation of PGAM5 signaling through regulation of the interaction between the MM and the phosphatase domain. Compounds mimicking this binding event could be of potential therapeutic interest in the treatment of stroke, myocardial infarction, and other diseases by stimulating the mitophagy-promoting functions of PGAM5.

## Methods

### Protein expression and purification

Wild-type full-length PGAM5 cDNA (corresponding to the PGAM5 long isoform (PGAM5L), residues 1–289) was purchased from Addgene. For bacterial expression and purification, the Δ90 PGAM5 (residues 91–289) and Δ48 PGAM5 (residues 49–289) were cloned into the pET28a expression vector (Novagen). These constructs were N-terminally tagged with hexahistidine (6× -HIS) followed by a linker sequence encoding a TEV protease cleavage site (ENLYFQS). Full-length and ∆23 (residues 24–289) PGAM5 variants for mammalian cell expression and imaging were cloned into the pcDNA3.1+ vector (Invitrogen). These constructs contained an internal myc-tag (EQKLISEEDL) inserted between residue 42 and 43 of the PGAM5 sequence. Mutations were introduced using either QuickChange mutagenesis or through Gibson assembly^57^. Primers used for mutagenesis are as follows: H105A (Forward: 5′-acatcttcctcatcagggcttcccagtaccacgtgg-3′ Reverse: 5′-ccacgtggtactgggaagccctgatgaggaagatgt-3′), Y198E (F: 5′-gctccgtcttcctcatactgcacagcttccggc-3′ R: 5′-gccggaagctgtgcagtatgaggaagacggagc-3′), R65A (F: 5′-agagacagtggttctgccctgtcccagttggg-3′ R: 5′-cccaactgggacagggcagaaccactgtctct-3′), F244E (F: 5′-gccagccttcaggaggctcctgcagtgctctgcac-3′ R: 5′-gtgcagagcactgcaggagcctcctgaaggctggc-3′), R288E (F: 5′-cctcccgacaagatcactgaatcctgagatccgaattc-3′ R: 5′-gaattcggatctcaggattcagtgatcttgtcgggagg-3′). Mutation of WT A4 was done by sequential mutation of W58A and D59A and followed by W62A and D63A. W58A/D59A (F: 5′- gtcccagttgggggccgcgacaccggggccc-3′ R: 5′-gggccccggtgtcgcggcccccaactgggac-3′), W62A/D63A (F: 5′-gtggttctcgcctggccgcgttggggtcccaga-3′ R: 5′-tctgggaccccaacgcggccaggcgagaaccac-3′).

Y92A (F: 5′-cgtggccttggctttggcgtggtccagcttggac-3′ R: 5′-gtccaagctggaccacgccaaagccaaggccacg-3′), D111A (F: 5′-cccagtaccacgtggccggctccctggagaag-3′ R: 5′-cttctccagggagccggccacgtggtactggg-3′), K191A (F: 5′-tgcacagcttccggcgcccaatgagacacggg-3′ R: 5′-cccgtgtctcattgggcgccggaagctgtgca-3′), R218A/E220A (F: 5′-gtaactgtcctccgcctgcgcggc-0 = atctgcgcgg-3′ R: 5′-ccgcgcagatgccgcgcaggcggaggacagttac-3′).

For recombinant protein expression, BL21 DE3 pLysS *Escherichia coli* cells transformed with pET28a-PGAM5 constructs were cultured in Luria Broth media supplemented with kanamycin (30 µg/mL) at 37 °C until 0.4 optical density at 600 nm was reached. Protein expression was induced with 0.2 mM IPTG and cells were cultured for additional 12 h at 25 °C. Following collection by centrifugation at 5000×*g*, cell pellets were resuspended in Lysis Buffer (20 mM Tris-Cl pH 8.0, 500 mM NaCl, 0.5 mM TCEP, 5% glycerol, 20 mM Imidazole pH 8.0 supplemented with EDTA-free protease inhibitors (Roche)). Cells were lysed by sonication at 25% of max power in cycles of 4 s on and 2 s off for 5 min. Lysates were clarified by centrifugation at 18,000×*g* and loaded by gravity flow on HiTrap Ni-NTA resin (GE Healthcare). Protein was eluted using Elution Buffer (20 mM Tris-Cl pH 8.0, 500 mM NaCl, 0.5 mM TCEP, 5% glycerol, 200 mM Imidazole pH 8.0) and exchanged into buffer containing 20 mM Tris-Cl pH 8.0, 150 mM NaCl, and 0.5 mM TCEP using a 10 kDa cutoff concentrator (Millipore). The sample was further purified by SEC using a Superose 6 10/300 GL Increase column (GE Healthcare) equilibrated in buffer containing 20 mM Tris-Cl pH 8.0, 150 mM NaCl and 0.5 mM TCEP. Salt titration experiments were performed by SEC in buffer conditions containing 20 mM Tris-Cl pH 8.0, 0.5 mM TCEP and concentrations of NaCl ranging from 150 mM to 1 M.

### Peptides

The ASK1 phosphopeptide (NFEDH(pS)APPSP) and the PGAM5 MM peptide (GPGVWDPNWDRREP)^33^, were purchased at a purity of 95% or greater (Elim Biopharma). Peptides were resuspended in 20 mM Tris-Cl pH 7.5, aliquoted, and flash frozen in liquid nitrogen prior to storage at −80 °C.

### Negative-stain sample preparation and data acquisition

Homemade grids were prepared for negative-stain experiments by depositing a thin plastic layer on the surface of 400-mesh copper grids (Ted Pella) floated on 2% amyl acetate (EMS)^58^. Air-dried grids were then coated with ~3 nm of continuous carbon film, negative glow discharged, and loaded with 2.5 µL of purified PGAM5 (~0.01–0.1 mg/mL). Following a 30 s incubation, grids were briefly dipped in two drops of sample buffer and one drop of 0.75% uranyl formate staining solution with blotting performed between drops by touching the edge of the grid briefly to the surface of Whatman 1 filter paper. Sample grids were dipped in a second drop of stain for 30 s, blotted on filter paper, and air dried prior to imaging. Images were collected using a 120 kV Tecnai T12 (FEI) microscope equipped with a Gatan 4× 4 K CCD camera at 52 K magnification corresponding to a pixel size of 2.21 Å.

### Cryo-EM sample preparation, data acquisition, and processing

For cryo-EM experiments, we first screened grid type and protein buffer to identify optimal conditions for data collection. We observed significant orientation bias as well as aggregation on traditional Quantifoil 1.2/1.3 400-mesh spacing gold grids (Electron Microscopy Sciences) that could not be improved by changes in buffer conditions (i.e., varying salt concentration, addition of glycerol, addition of low concentrations of detergent). While aggregation was somewhat alleviated by sample application on C-flat 1.2/1.3 holey thick carbon grids (Electron Microscopy Sciences), sample quality appeared to be the best for 3 μL of purified PGAM5 sample (at 0.10 mg/mL) in ring stabilization buffer (20 mM Tris-Cl pH 8.0, 600 mM NaCl, 0.5 mM TCEP) applied to freshly glow-discharged Lacey Formvar/Carbon 400-mesh continuous carbon coated copper grids (Ted Pella) placed inside a Vitrobot Mark IV (FEI) equilibrated to 4 °C and 100% humidity. Following a 10 s incubation, grids were blotted between Whatman 1 paper for 6 s, and plunge-frozen in liquid ethane. Images were recorded using a 300 kV Titan Krios microscope (FEI) equipped with a K2 Summit detector (Gatan) operated in super-resolution mode (UC Berkeley). Totally, 6543 images were collected at a nominal magnification of 22.5 K, corresponding to a pixel size of 0.53 Å. Dose-fractionated movies were acquired at an electron flux of 1.8 e^−1^/A^2^ over 7.75 s with 0.25 s exposures per frame (31 frames total), corresponding to a total dose of 55 e^−^^1^/Å^2^. Images were recorded with a defocus range of −1 to 2.5 μm.

Frame-based motion correction, 2× binning, and dose weighting were performed on dose-fractionated image stacks using MotionCor2^59^ resulting in one integrated image per stack with a pixel size of 1.07 Å. The contrast transfer function was estimated from motion corrected, nondose-weighted images using GCTF version 1.06^60^. From an initial subset of micrographs, approximately 1000 particles were manually picked and subject to likelihood-based 2D classification in Relion 2.0^61^ to generate templates for automated particle selection using Gautomatch version 0.55^60^. In total, 131,396 particles were extracted at a box size of 190 pixels with 2.14 Å/pixel and subject to 2D classification to eliminate particles of poor quality resulting in a dataset of 22,422 particles corresponding to orientations in which C6 symmetry of the dodecameric assembly of ∆48 PGAM5 was observed. The final selected particles were re-centered and re-extracted at full pixel size to generate the class average shown in Fig. 2a. Data collection details are reported in Supplementary Table 1.

### X-ray crystallography

The ∆90 H105A PGAM5 construct was crystallized using hanging drop vapor diffusion at room temperature in the presence of the ASK1 phosphopeptide alone or together with the MM peptide. The electron density corresponding to the ASK1 peptide could not be resolved in any of the structures. For crystallization of ∆90 H105A PGAM5 with the ASK1 peptide, protein was concentrated to 5 mg/mL and incubated with the peptide on ice at a 5:1 molar ratio of peptide:protein. Crystals were grown in a 2 μL drop (1 μL protein + 1 μL reservoir solution) against a reservoir solution comprised of 100 mM HEPES pH 7.5, 200 mM MgCl_2_ and 30% w/v PEG 400. For crystallization of the ∆90 H105A PGAM5 in complex with the MM peptide, protein at a concentration of 7.5 mg/mL was incubated on ice with 3:3:1 molar ratio of ASK1 peptide:MM peptide:protein. Crystals were grown in 2 μL drops (1 μL protein + 1 μL reservoir solution) equilibrated against reservoir solution containing 100 mM Tris-Cl pH 7.0, 200 mM MgCl_2_ and 10% w/v PEG 8000. Crystals from both conditions were cryoprotected with Paratone-N oil and flash-cooled to 100 K in liquid nitrogen.

X-ray diffraction data were collected at 1.111 Å, 77 K at the ALS 8.3.1. beamline (Berkeley, CA) on a Pilatus 6M detector. Data were indexed with XDS^62^, scaled in Aimless^63^ and phases determined in Phaser^64^. ∆90 PGAM5 H105A crystals diffracted to 1.7 Å in space group P2_1_2_1_2_1_ and with unit cell dimensions: *a* = 71.0 Å, *b* = 72.0 Å, *c* = 81.9 Å, *α* = *β* = *γ* = 90° and with two molecules in the asymmetric unit. A solution was generated by molecular replacement using one copy of PDB 3MXO as the search model. Crystals of ∆90 PGAM5 H105A/MM diffracted to 2.6 Å in the space group *P*2_1_2_1_2_1_ with unit cell dimensions: *a* = 49.3 Å, *b* = 242.5 Å, *c* = 272.5 Å, *α* = *β* = *γ* = 90° and with twelve molecules in the asymmetric unit. The structure was solved by molecular replacement using a single monomer of PDB 5MUF and searching for 12 copies in the asymmetric unit. Refinement was performed in Phenix^65^ with iterative rounds of model building in Coot^66^. Model quality was assessed with MolProbity^67^ and coordinates deposited in the RCSB PDB databank under the PDB codes 6CNI (∆90 PGAM5 H105A) and 6CNL (∆90 PGAM5 H105A/MM). Detailed statistics for data collection and structure refinement are reported in Table 1.

### Phosphatase activity assay

Phosphatase assays were performed at room temperature for wild type and mutant constructs of 6xHIS-∆48 PGAM5 (A4, R65A, H105A, Y198E, F244E, R269E, and R288E). ASK1 phosphopeptide was serially diluted (0–200 µM) into reaction buffer (20 mM Tris-Cl pH 8.0, 150 mM NaCl and 0.5 mM TCEP) and the assay was started by the addition of PGAM5 (20 nM). The reaction was quenched at various time points with the addition of malachite green (Promega) and the total free phosphate in solution measured as the absorbance at 620 nm. Initial rates and Michaelis–Menten kinetic parameters were calculated in Prism (GraphPad) and are reported in Table 2.

### Subcellular fractionation

HEK293T cells (ATCC) cultured under normal conditions were treated with CCCP (50 µM) (Carbonyl cyanide 3-chlorophenylhydrazone, Sigma Aldrich) prepared in complete media for 4 h prior to harvest. Following treatment, subcellular fractionation of cellular cytoplasm and mitochondria was performed by resuspending harvested cells first with plasma membrane (PM) permeabilization buffer (20 mM HEPES, 100 mM Sucrose, 2.5 mM MgCl_2_, 100 mM KCl, 10 mM DTT, 0.025% digitonin, pH 7.4) supplemented with DNase (Roche) and complete protease inhibitors without EDTA (Roche). Following incubation at 4 °C for 30 min with end over end mixing, membrane fractions were separated from cytosol by centrifugation at 4 °C and 13,000×*g* for 10 min. Supernatant was collected as the cytosolic (cyto) fraction. The remaining pellet was washed twice by resuspending in cold PBS and centrifugation at 4 °C and 600×*g* for 5 min in between washes. Resuspended mitochondrial membranes were then solubilized in a volume of mitochondrial permeabilization buffer (20 mM Bis–Tris, 50 mM NaCl, 10% glycerol, 10 mM DTT, 2% digitonin, pH 7.4, supplemented with protease inhibitors) equivalent to the volume of PM buffer used and incubated with end over end mixing for 30 min at 4 °C prior to one final centrifugation at 4 °C and 13,000×*g* for 10 min. Supernatant from this fraction was collected as the mitochondrial (mito) fraction. Samples were prepared for sodium dodecyl sulfate polyacrylamide gel electrophoresis (SDS-PAGE) loading controls and BN-PAGE by adding equivalent volumes of either standard SDS-PAGE loading dye or 10× BN-PAGE loading dye (100 mM Bis–Tris pH 7.0, 500 mM 6-amino-nexamoic acid, and 5% Coomassie Blue R-250), respectively. Samples for SDS-PAGE were normalized for concentration and subject to immunoblotting with antibodies against PGAM5 (1:3000) (kind gift from Z. Wang), β-tubulin (1:2000) (Cell Signaling Technologies, Inc., 2128S), and Tom20 (1:100) (Santa Cruz Biotechnologies, Inc., sc-17764). Uncropped scans of blots shown in Fig. 6a are included in Supplementary Figure 6.

### Blue Native-PAGE

BN-PAGE was performed for equivalent volumes of the total fractionation sample supplemented with BN-PAGE loading dye^68,69^. Samples were loaded onto a 4–16% Native-PAGE gel (Invitrogen) and electrophoresed under 8 mA of constant current in blue cathode buffer (Invitrogen) until the dye front had migrated approximately one-third of the way through the resolving gel. Next, the blue cathode buffer was replaced with clear anode buffer (Invitrogen) and electrophoresis continued at 150 V for 60 min, then increased to 250 V for the remainder of the experiment (45 min). Prior to transfer to polyvinylidene fluoride (PVDF) (Millipore) and immunoblotting, the native gels was soaked for 10 min in tris-glycine transfer buffer supplemented with 20% methanol and 0.05% SDS. Following the transfer, the PVDF membrane was completely destained in 100% methanol, then equilibrated in blocking buffer (5% w/v milk prepared in TBST buffer) prior to immunoblotting with anti-PGAM5 antibody.

### Confocal imaging

Monkey kidney COS7 cells (obtained from J. Kuriyan, UC Berkeley) were cultured at 37 °C in 5% CO_2_ with DMEM supplemented with 10% fetal bovine serum (FBS), and 1% penicillin–streptomycin (Thermo Fisher). Mouse embryonic fibroblasts (obtained from D. Chan, Caltech) were cultured at 37 °C in 5% CO_2_ with DMEM supplemented with 10% FBS, 100 μM nonessential amino acids and 1% penicillin–streptomycin (Thermo Fisher). For imaging, COS7 and MEF cells were plated at a density of 0.9 × 10^5^ cells per well in a six-well plate (Corning) on acid-washed glass coverslips and grown for 16 h at 37 °C. Cells were then transfected with 500 ng of full-length or cleaved (Δ23) PGAM5 using Lipofectamine 3000 (Invitrogen) and incubated for 24 h at 37 °C. For nocodazole treatments, relevant cells were incubated with 10 μM nocodazole in media for 45 min at 37 °C. Following transfection and drug treatment, cells were washed with cold PBS, and fixed with 3.7% formaldehyde for 1 h at room temperature. After three rinses with PBS, cells were permeabilized with 0.1% Triton X-100 for 5 min, then washed three times with PBS. Permeabilized cells were incubated with the blocking buffer (1% BSA in PBS) for 5 min at room temperature, then for 1 h at 37 °C with blocking buffer supplemented with primary antibodies. Those included anti-myc (mouse, 1:6000 dilution in blocking buffer) (Millipore, 05-724), anti-Tom20 (rabbit, 1:250) (Santa Cruz Biotechnology, sc-17764), anti-β-tubulin (rabbit, 1:25) (Cell Signaling Technologies, 2128S), anti-β-tubulin (rabbit, 1:250) (Abcam, Ab179513), or Alexa Fluor-647 Phalloidin (1:20) (Thermo Fischer, A22287). For Tim23 labeling, blocking buffer contained 10% FBS and primary labeling used Anti-Tim23 antibody (rabbit, 1:250) (Proteintech, 11123-1-AP). Coverslips were washed three times with PBS and incubated with respective secondary antibodies: FITC-488 (rabbit, 1:500) (Invitrogen, A-11034) and Alexa-647 (mouse, 1:500) (Invitrogen, A327728) for 1 h at 37 °C in the dark. Following three washes with PBS, samples were incubated with the nuclei stain DAPI (1:1000 dilution in water) (Thermo Fischer, D1306) for 5 min at room temperature. Following three final water washes to remove excess DAPI, coverslips were mounted using ProLong Gold (Invitrogen) and dried overnight at room temperature in the dark. Imaging was done using a Nikon TIRF microscope with a Yokagawa CSU-X1 spinning disk confocal consisting of 405, 491, 561 and 640 nm lasers with the use of a 60x objective. Data were processed using FIJI v1.0 (55).

### Structure illumination imaging

COS7 cells for SIM imaging were cultured, transfected, and prepared for imaging as for confocal microscopy. Slides were imaged on an N-SIM (OMX microscope with 405, 488, 561, and 640 nm lasers) using an Apo TIRF 100× objective with 1.56 oil in 3D mode and standard operating procedure. Image reconstruction was performed in SoftWoRx v6.5.2 (GE Healthcare).

### STED imaging

COS7 cells for STED imaging were cultured, transfected, and prepared for imaging as for confocal and SIM microscopy, however, using different secondary antibodies: secondary Alexa-594 (rabbit, 1:500) (Invitrogen, R37117) and Alexa-647 (mouse, 1:500) (Invitrogen) antibodies. Images were acquired using a Leica STED SP8 microscope (equipped with a white light laser, a 775 nm depletion laser and 100× objective HC PL APO 100×/1.40 OIL) using standard operating procedure. Images were processed in FIJI v1.0^70^.

### Reporting Summary

Further information on experimental design is available in the Nature Research Reporting Summary linked to this Article.

## Supplementary Information

Supplementary Information

Reporting Summary

## Data Availability

Coordinates have been deposited in the Protein Data Bank with the accession number 6CNI (∆90 PGAM5 H105A) and 6CNL (∆90 PGAM5 H105A/MM). Data supporting the findings of this manuscript are available from the corresponding author upon reasonable request.

## References

[1] Rigden DJ (2008). The histidine phosphatase superfamily: structure and function. Biochem J..

[2] Jedrzejas MJ (2000). Structure, function, and evolution of phosphoglycerate mutases: comparison with fructose-2,6-bisphosphatase, acid phosphatase, and alkaline phosphatase. Prog. Biophys. Mol. Biol..

[3] Takeda K (2009). Mitochondrial phosphoglycerate mutase 5 uses alternate catalytic activity as a protein serine/threonine phosphatase to activate ASK1. Proc. Natl Acad. Sci. USA.

[4] Panda S (2016). Identification of PGAM5 as a mammalian protein histidine phosphatase that plays a central role to negatively regulate CD4(+) T cells. Mol. Cell.

[5] Lo SC, Hannink M (2008). PGAM5 tethers a ternary complex containing Keap1 and Nrf2 to mitochondria. Exp. Cell Res.

[6] Sekine S (2012). Rhomboid protease PARL mediates the mitochondrial membrane potential loss-induced cleavage of PGAM5. J. Biol. Chem..

[7] Lu W (2014). Genetic deficiency of the mitochondrial protein PGAM5 causes a Parkinson’s-like movement disorder. Nat. Commun..

[8] Saita S (2017). PARL mediates Smac proteolytic maturation in mitochondria to promote apoptosis. Nat. Cell Biol..

[9] Zhuang M, Guan S, Wang H, Burlingame AL, Wells JA (2013). Substrates of IAP ubiquitin ligases identified with a designed orthogonal E3 ligase, the NEDDylator. Mol. Cell.

[10] Wai T (2016). The membrane scaffold SLP2 anchors a proteolytic hub in mitochondria containing PARL and the i-AAA protease YME1L. EMBO Rep..

[11] Bernkopf, D.B. et al. Pgam5 released from damaged mitochondria induces mitochondrial biogenesis via Wnt signaling. *J. Cell Biol.* **217**, 1383–1394 (2018). 10.1083/jcb.201708191 PMC5881504 29438981

[12] Lo SC, Hannink M (2006). PGAM5, a Bcl-XL-interacting protein, is a novel substrate for the redox-regulated Keap1-dependent ubiquitin ligase complex. J. Biol. Chem..

[13] O’Mealey GB (2017). A PGAM5-KEAP1-Nrf2 complex is required for stress-induced mitochondrial retrograde trafficking. J. Cell Sci..

[14] Imai Y (2010). The loss of PGAM5 suppresses the mitochondrial degeneration caused by inactivation of PINK1 in Drosophila. PLoS Genet.

[15] Matsuda N (2010). PINK1 stabilized by mitochondrial depolarization recruits Parkin to damaged mitochondria and activates latent Parkin for mitophagy. J. Cell Biol..

[16] Vives-Bauza C (2010). PINK1-dependent recruitment of Parkin to mitochondria in mitophagy. Proc. Natl Acad. Sci. USA.

[17] Geisler S (2010). PINK1/Parkin-mediated mitophagy is dependent on VDAC1 and p62/SQSTM1. Nat. Cell Biol..

[18] Narendra DP (2010). PINK1 is selectively stabilized on impaired mitochondria to activate Parkin. PLoS Biol..

[19] Chen G (2014). A regulatory signaling loop comprising the PGAM5 phosphatase and CK2 controls receptor-mediated mitophagy. Mol. Cell.

[20] Gispert S (2009). Parkinson phenotype in aged PINK1-deficient mice is accompanied by progressive mitochondrial dysfunction in absence of neurodegeneration. PLoS One.

[21] Glasl L (2012). Pink1-deficiency in mice impairs gait, olfaction and serotonergic innervation of the olfactory bulb. Exp. Neurol..

[22] Kitada T (2007). Impaired dopamine release and synaptic plasticity in the striatum of PINK1-deficient mice. Proc. Natl Acad. Sci. USA.

[23] Valente EM (2004). Hereditary early-onset Parkinson’s disease caused by mutations in PINK1. Science.

[24] Wang Z, Jiang H, Chen S, Du F, Wang X (2012). The mitochondrial phosphatase PGAM5 functions at the convergence point of multiple necrotic death pathways. Cell.

[25] Ishida Y (2012). Prevention of apoptosis by mitochondrial phosphatase PGAM5 in the mushroom body is crucial for heat shock resistance in *Drosophila melanogaster*. PLoS One.

[26] Moriwaki K (2016). The mitochondrial phosphatase PGAM5 is dispensable for necroptosis but promotes inflammasome activation in macrophages. J. Immunol..

[27] Murphy JM (2013). The pseudokinase MLKL mediates necroptosis via a molecular switch mechanism. Immunity.

[28] Remijsen Q (2014). Depletion of RIPK3 or MLKL blocks TNF-driven necroptosis and switches towards a delayed RIPK1 kinase-dependent apoptosis. Cell Death Dis..

[29] Moujalled DM, Cook WD, Murphy JM, Vaux DL (2014). Necroptosis induced by RIPK3 requires MLKL but not Drp1. Cell Death Dis..

[30] He GW (2017). PGAM5-mediated programmed necrosis of hepatocytes drives acute liver injury. Gut.

[31] Xu W (2015). Bax-PGAM5L-Drp1 complex is required for intrinsic apoptosis execution. Oncotarget.

[32] Lu W (2016). Mitochondrial protein PGAM5 regulates mitophagic protection against cell necroptosis. PLoS One.

[33] Wilkins JM, McConnell C, Tipton PA, Hannink M (2014). A conserved motif mediates both multimer formation and allosteric activation of phosphoglycerate mutase 5. J. Biol. Chem..

[34] Chaikuad A (2017). Structures of PGAM5 provide insight into active site plasticity and multimeric assembly. Structure.

[35] O’Mealey, G.B. et al. A PGAM5-KEAP1-Nrf2 complex is required for stress-induced mitochondrial retrograde trafficking. *J. Cell Sci.* **130**, 3467–3480 (2017). 10.1242/jcs.203216 PMC5665445 28839075

[36] Ingerman E (2005). Dnm1 forms spirals that are structurally tailored to fit mitochondria. J. Cell Biol..

[37] Osellame LD (2016). Cooperative and independent roles of the Drp1 adaptors Mff, MiD49 and MiD51 in mitochondrial fission. J. Cell Sci..

[38] Ishihara N (2009). Mitochondrial fission factor Drp1 is essential for embryonic development and synapse formation in mice. Nat. Cell Biol..

[39] Wakabayashi J (2009). The dynamin-related GTPase Drp1 is required for embryonic and brain development in mice. J. Cell Biol..

[40] Stratton M (2014). Activation-triggered subunit exchange between CaMKII holoenzymes facilitates the spread of kinase activity. Elife.

[41] Kalia R (2018). Structural basis of mitochondrial receptor binding and constriction by DRP1. Nature.

[42] Engel A, Walter P (2008). Membrane lysis during biological membrane fusion: collateral damage by misregulated fusion machines. J. Cell Biol..

[43] Papanicolaou KN, Phillippo MM, Walsh K (2012). Mitofusins and the mitochondrial permeability transition: the potential downside of mitochondrial fusion. Am. J. Physiol. Heart Circ. Physiol..

[44] Katsov K, Muller M, Schick M (2006). Field theoretic study of bilayer membrane fusion: II. Mechanism of a stalk-hole complex. Biophys. J..

[45] Marrink SJ, Mark AE (2003). The mechanism of vesicle fusion as revealed by molecular dynamics simulations. J. Am. Chem. Soc..

[46] Muller M, Katsov K, Schick M (2002). New mechanism of membrane fusion. J. Chem. Phys..

[47] Noguchi H, Takasu M (2001). Fusion pathways of vesicles: a Brownian dynamics simulation. J. Chem. Phys..

[48] Reichert AS, Neupert W (2002). Contact sites between the outer and inner membrane of mitochondria-role in protein transport. Biochim Biophys. Acta.

[49] Perkins GA, Frey TG (2000). Recent structural insight into mitochondria gained by microscopy. Micron.

[50] Strauss M, Hofhaus G, Schroder RR, Kuhlbrandt W (2008). Dimer ribbons of ATP synthase shape the inner mitochondrial membrane. EMBO J..

[51] Nunnari J, Suomalainen A (2012). Mitochondria: in sickness and in health. Cell.

[52] Claypool SM, Oktay Y, Boontheung P, Loo JA, Koehler CM (2008). Cardiolipin defines the interactome of the major ADP/ATP carrier protein of the mitochondrial inner membrane. J. Cell Biol..

[53] Paumard P (2002). The ATP synthase is involved in generating mitochondrial cristae morphology. EMBO J..

[54] Barbot M (2015). Mic10 oligomerizes to bend mitochondrial inner membranes at cristae junctions. Cell Metab..

[55] Fu TM (2016). Cryo-EM structure of caspase-8 tandem DED filament reveals assembly and regulation mechanisms of the death-inducing signaling complex. Mol. Cell.

[56] Li J (2012). The RIP1/RIP3 necrosome forms a functional amyloid signaling complex required for programmed necrosis. Cell.

[57] Gibson DG (2009). Enzymatic assembly of DNA molecules up to several hundred kilobases. Nat. Methods.

[58] Booth, D. S., Avila-Sakar, A. & Cheng, Y. Visualizing proteins and macromolecular complexes by negative stain EM: from grid preparation to image acquisition. *J. Vis. Exp.* **58**, e3227 (2011). 10.3791/3227 PMC3369646 22215030

[59] Zheng SQ (2017). MotionCor2: anisotropic correction of beam-induced motion for improved cryo-electron microscopy. Nat. Methods.

[60] Zhang K (2016). Gctf: Real-time CTF determination and correction. J. Struct. Biol..

[61] Kimanius, D., Forsberg, B. O., Scheres, S. H. & Lindahl, E. Accelerated cryo-EM structure determination with parallelisation using GPUs in RELION-2. *eLife* **5**, e18722 (2016). 10.7554/eLife.18722 PMC5310839 27845625

[62] Kabsch W (2010). Integration, scaling, space-group assignment and post-refinement. Acta Crystallogr D Biol. Crystallogr..

[63] Otwinowski Z, Minor W (1997). Processing of X-ray diffraction data collected in oscillation mode. Methods Enzymol..

[64] McCoy AJ (2007). Phaser crystallographic software. J. Appl. Crystallogr..

[65] Adams PD (2010). PHENIX: a comprehensive Python-based system for macromolecular structure solution. Acta Crystallogr. D Biol. Crystallogr..

[66] Emsley P, Cowtan K (2004). Coot: model-building tools for molecular graphics. Acta Crystallogr. D Biol. Crystallogr..

[67] Chen VB (2010). MolProbity: all-atom structure validation for macromolecular crystallography. Acta Crystallogr. D Biol. Crystallogr..

[68] Wittig I, Braun HP, Schagger H (2006). Blue native PAGE. Nat. Protoc..

[69] Ma, S. et al. Assembly of the Bak apoptotic pore: a critical role for the Bak protein alpha6 helix in the multimerization of homodimers during apoptosis. *J. Biol. Chem*. **288**, 26027–26038 (2013). 10.1074/jbc.M113.490094 PMC3764807 23893415

[70] Schindelin J (2012). Fiji: an open-source platform for biological-image analysis. Nat. Methods.

[71] Baker NA, Sept D, Joseph S, Holst MJ, McCammon JA (2001). Electrostatics of nanosystems: application to microtubules and the ribosome. Proc. Natl Acad. Sci. USA.

