# Supplementary material for: Functional role of PGAM5 multimeric assemblies and their polymerization into filaments
Source: Nat Commun. 2019 Jan 31;10:531. doi: 10.1038/s41467-019-08393-w (PMC6355839; doi:10.1038/s41467-019-08393-w)
Supplement: Supplementary file 1 — Supplementary Information [file 41467_2019_8393_MOESM1_ESM.pdf]

Supplementary Information

Functional role of PGAM5 multimeric assemblies and their polymerization into filaments

Karen Ruiz[1,*], Tarjani M. Thaker[1,*], Christopher Agnew[1], Lakshmi Miller-Vedam[2], Raphael Trenker[1], Clara Herrera[3], Maria Ingaramo[4], Daniel Toso[5], Adam Frost[2,5,6], Natalia Jura[1, 7, **]

* These authors contributed equally to this work

** Correspondence should be addressed to N.J.

555 Mission Bay Blvd S, Rm 452W

San Francisco, CA 94158

Phone: 415-514-1133

Email: natalia.jura@ucsf.edu

Supplementary Figure 1

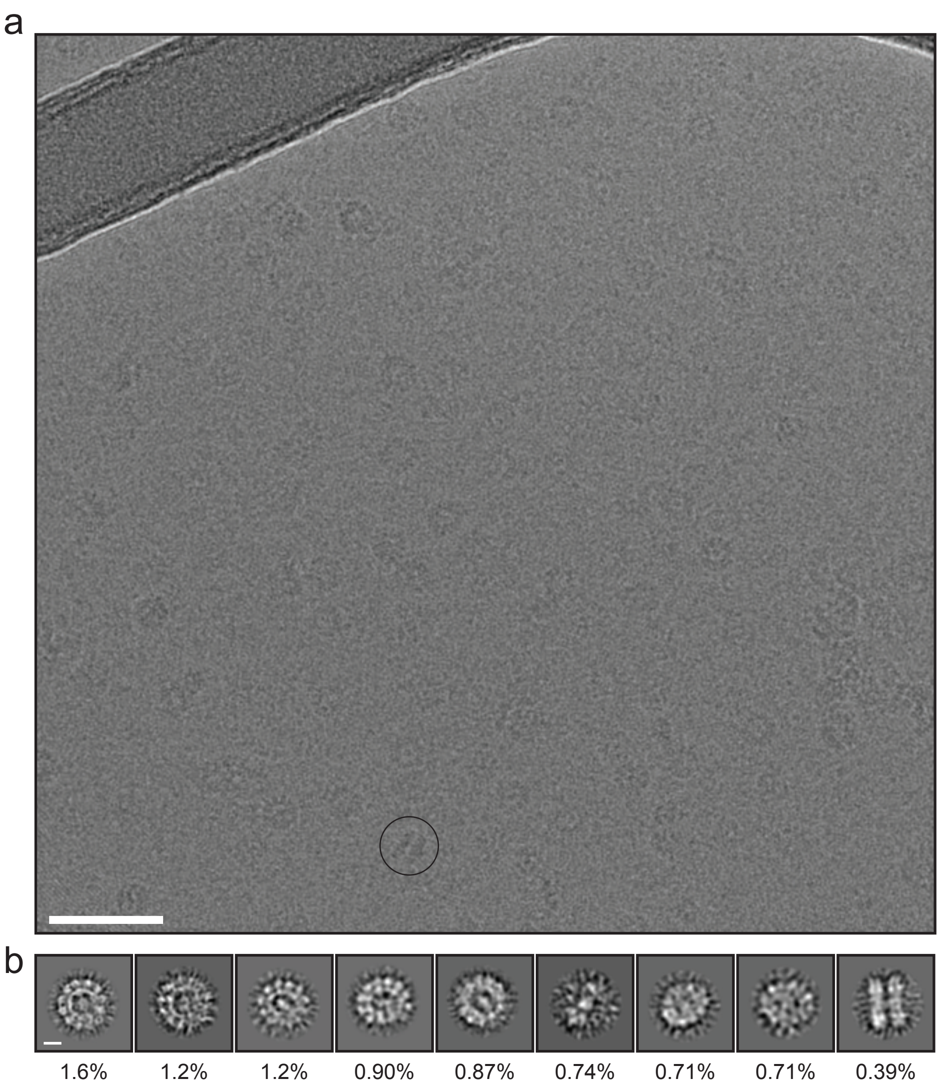

Supplementary Figure 1. Representative electron cryo-micrograph and 2D class averages of Δ48 PGAM5 dodecamer side views. **a,** Representative raw micrograph image of Δ48 PGAM5, with a representative side view of a dodecamer doublet circled on the image. **b,** Approximately 8.3% of the total number of particles picked from 6,543 micrographs corresponded to orientations other than the C6 symmetry 'top' view of the dodecamer and were subject to iterative rounds of 2D-classification into 9 classes as described in the methods section of the main text, revealing a single class in which the side view of a doublet of dodecamers stacked on the apparent planar face of the assembly (class 9 comprised of 542 particles) is observed. The scale bar in (b) corresponds to 5 nm.

Supplementary Figure 2

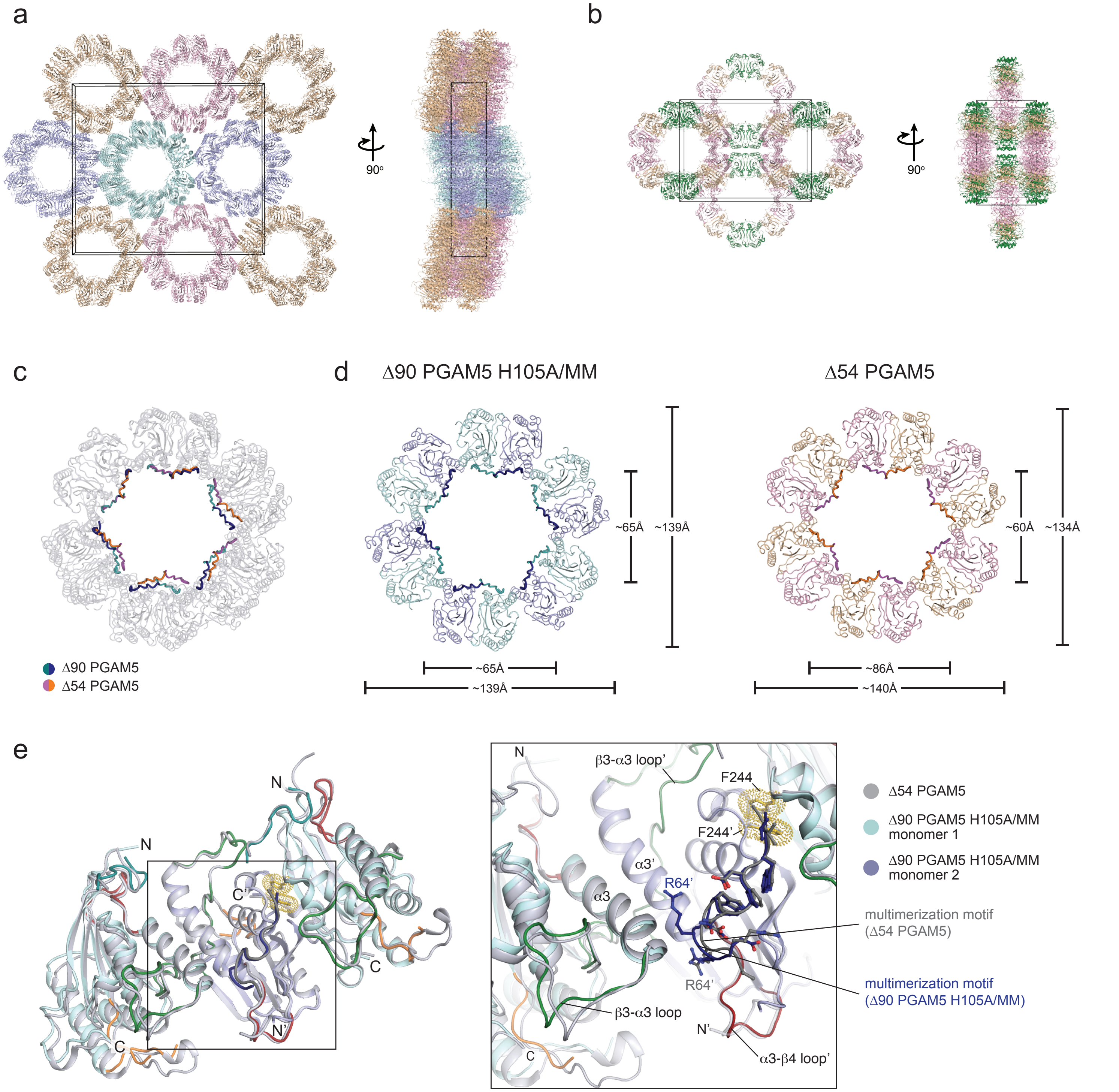

Supplementary Figure 2: Overview of dodecamer geometry in Δ90 PGAM5 H105A/MM compared to Δ54 PGAM5. Comparison of crystal packing in the structures of **a,** Δ90 PGAM5 H105A/MM and **b,** Δ54 PGAM5 (PDB: 5MUF). Molecules related by symmetry are colored the same in the respective lattices. **c,** Overlay of the dodecameric assemblies of Δ90 PGAM5 H105A/MM and Δ54 PGAM5, highlighting the differences in the geometry of the dodecamer lumen. **d,** Comparison of dodecamer dimensions in Δ90 PGAM5 H105A/MM (left panel) and Δ54 PGAM5 (right panel). **e,** Differences in the dimerization interface mediated by the α3 helices of two adjacent phosphatase domains are highlighted in the structures of Δ54 PGAM5 (grey) and Δ90 PGAM5 H105A/MM colored using the same color scheme as shown in Fig. 2 (monomer 1 in cyan; monomer 2 in light blue). A detailed view of the interface and residues within the multimerization motifs in the monomer 1 in both structures are shown as stick representation in the panel on the right.

Supplementary Figure 3

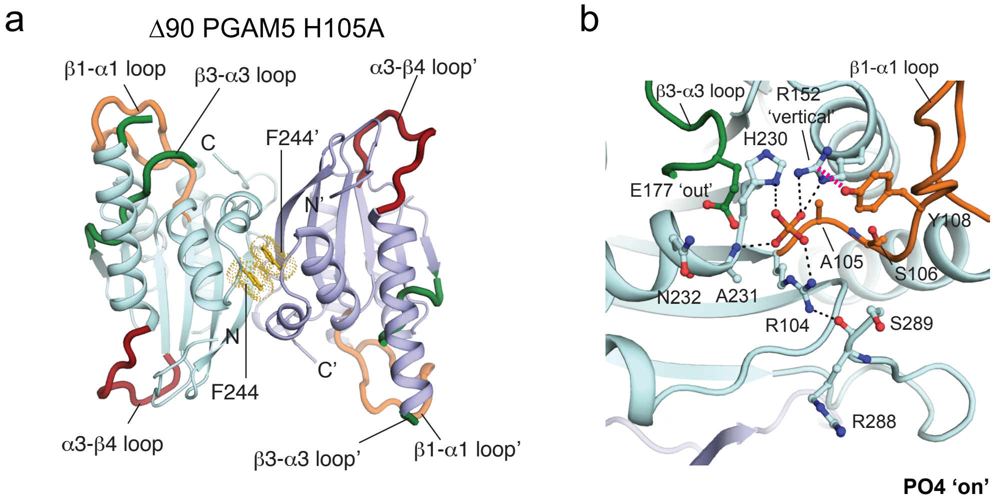

Supplementary Figure 3: Active site in the crystal structure of the Δ90 PGAM5 H105A phosphatase. **a,** The cartoon representation of the crystal structure of the Δ90 PGAM5 H105A phosphatase is shown using the same color assignment as in Fig. 3, with monomers 1 and 2 shown in cyan and light blue, respectively. The β1-α1 loops are colored in orange, the β3-α3 loops are colored in green, the α3-β4 loops are colored in red. The F244 residues in the constitutive dimer interface are colored in yellow. **b,** Detailed view of the residue positions in the active site in the crystal structure of the Δ90 PGAM5 H105A phosphatase depicting the coordination of a phosphate ion (PO4 'on').

Supplementary Figure 4

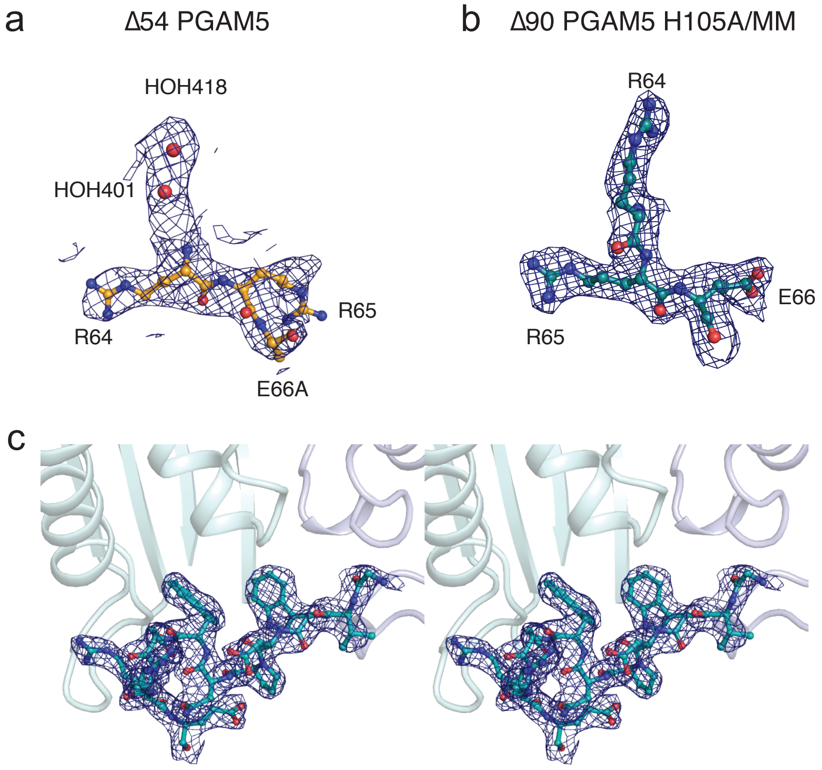

Supplementary Figure 4: Multimerization motif residue register and corresponding electron density. Differences in the electron density ($2F_o$-F_c maps contoured to 1σ), and subsequent amino acid assignment, of the multimerization motif region (residues 64 – 66) interacting with the phosphatase domain in **a,** Δ54 PGAM5 (PDB: 5MUF) and **b,** Δ90 PGAM5 H105A/MM. c) Stereo representation of the multimerization motif peptide (teal) associated with monomer 1 (cyan) in Δ90 PGAM5 H105A/MM, with corresponding $2F_o$-F_c electron density contoured to 1.0σ.

Supplementary Figure 5

a

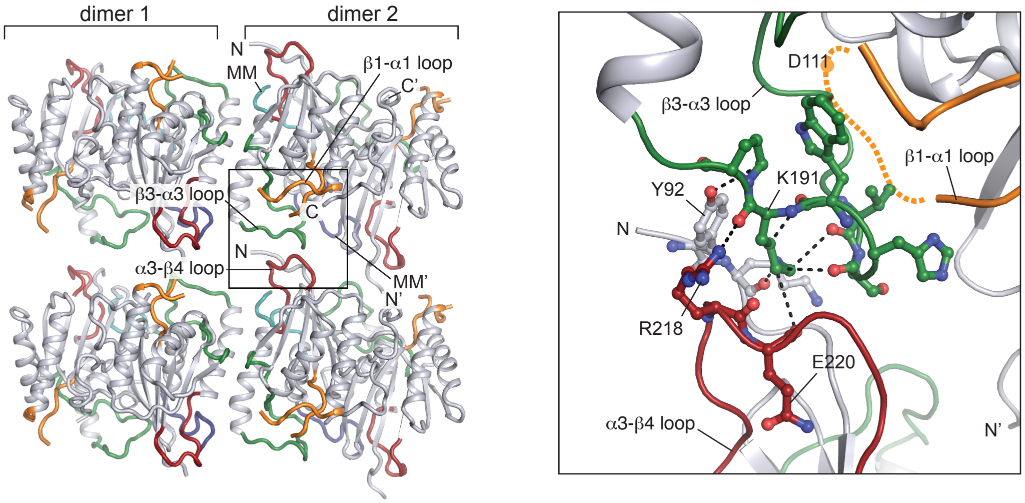

b

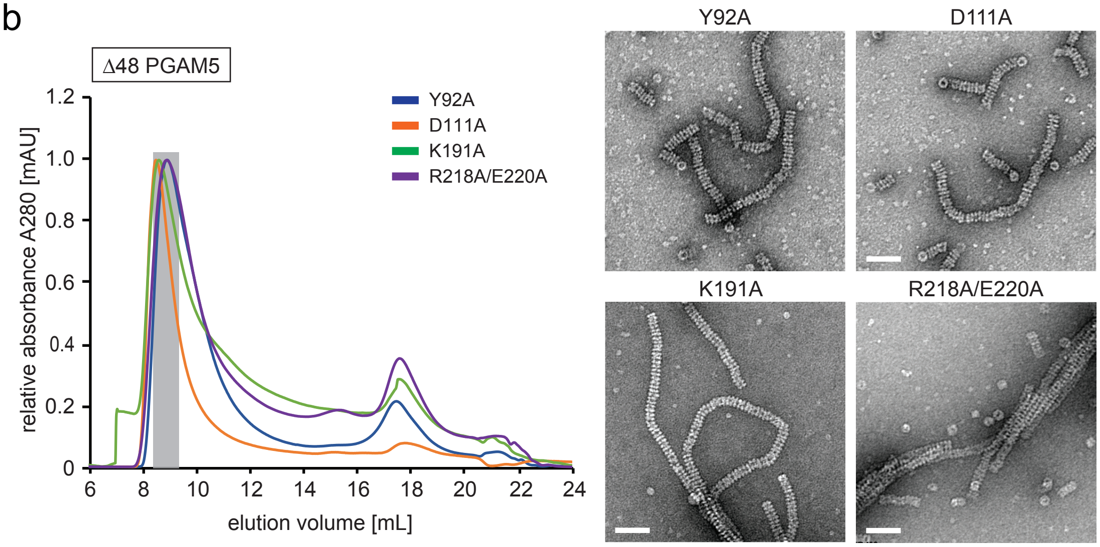

Supplementary Figure 5: Effect of mutations in the residues making direct contact at the crystallographic dodecamer stacking interface. a, Overview of the stacking interface between adjacent rings in the crystalline lattice of the Δ90 PGAM5 H105A/MM structure (left panel). Residues forming hydrogen bonding interactions at contact sites between rings are highlighted in the right panel. **b,** Elution profiles for Δ48 PGAM5 constructs carrying the indicated mutations of residues identified in (a) and purified by size exclusion chromatography (SEC) using a Superose 6 column (GE Healthcare) in buffer containing 150 mM NaCl. EM micrographs of negatively-stained samples of the PGAM5 mutants taken directly from the oligomer peaks observed during SEC runs are shown to the right.

Supplementary Figure 6

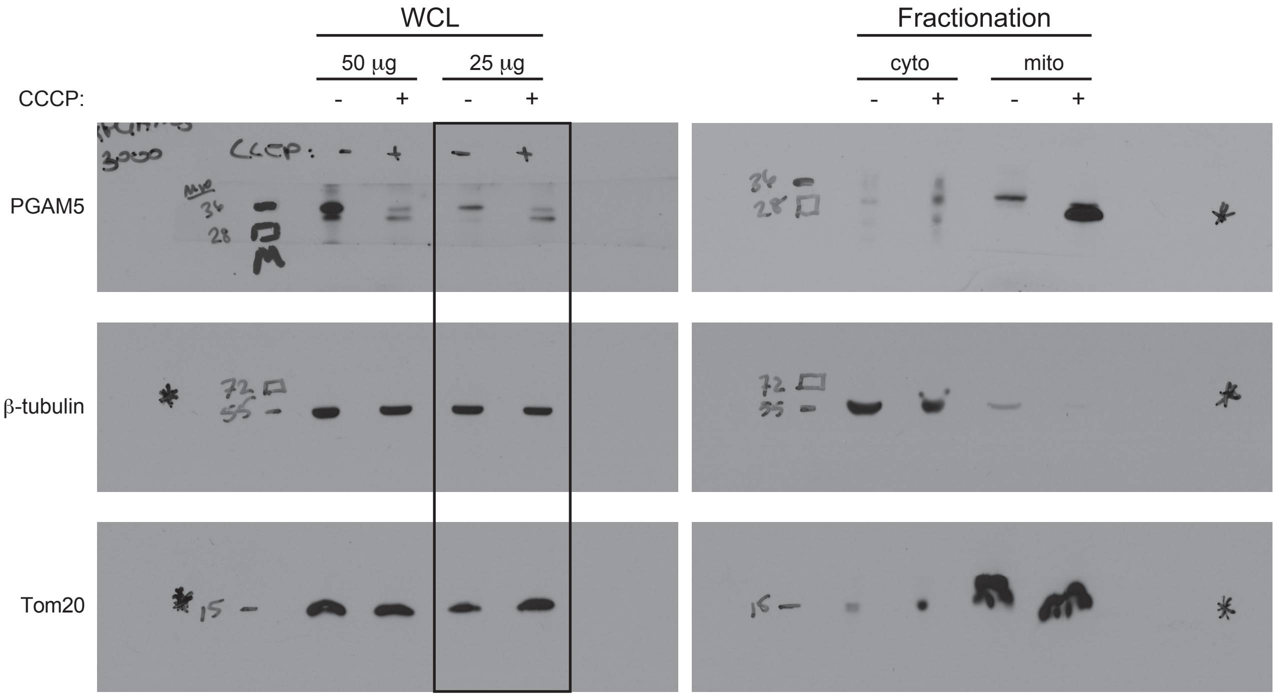

Supplementary Figure 6: Cleaved PGAM5 forms oligomers in cells. Uncropped blots corresponding to the data presented in Fig. 6a, showing the immunodetection of endogenous PGAM5 with anti-PGAM5 antibody, β-tubulin with anti-β-tubulin antibody, and Tom20 with anti-Tom20 antibody in HEK293T whole cell lysates (WCL), and in mitochondrial (mito), and cytoplasmic (cyto) fractions, 4 hours post CCCP treatment. WCL samples correspond to 25 μg of total protein loaded in each lane.

Supplementary Figure 7

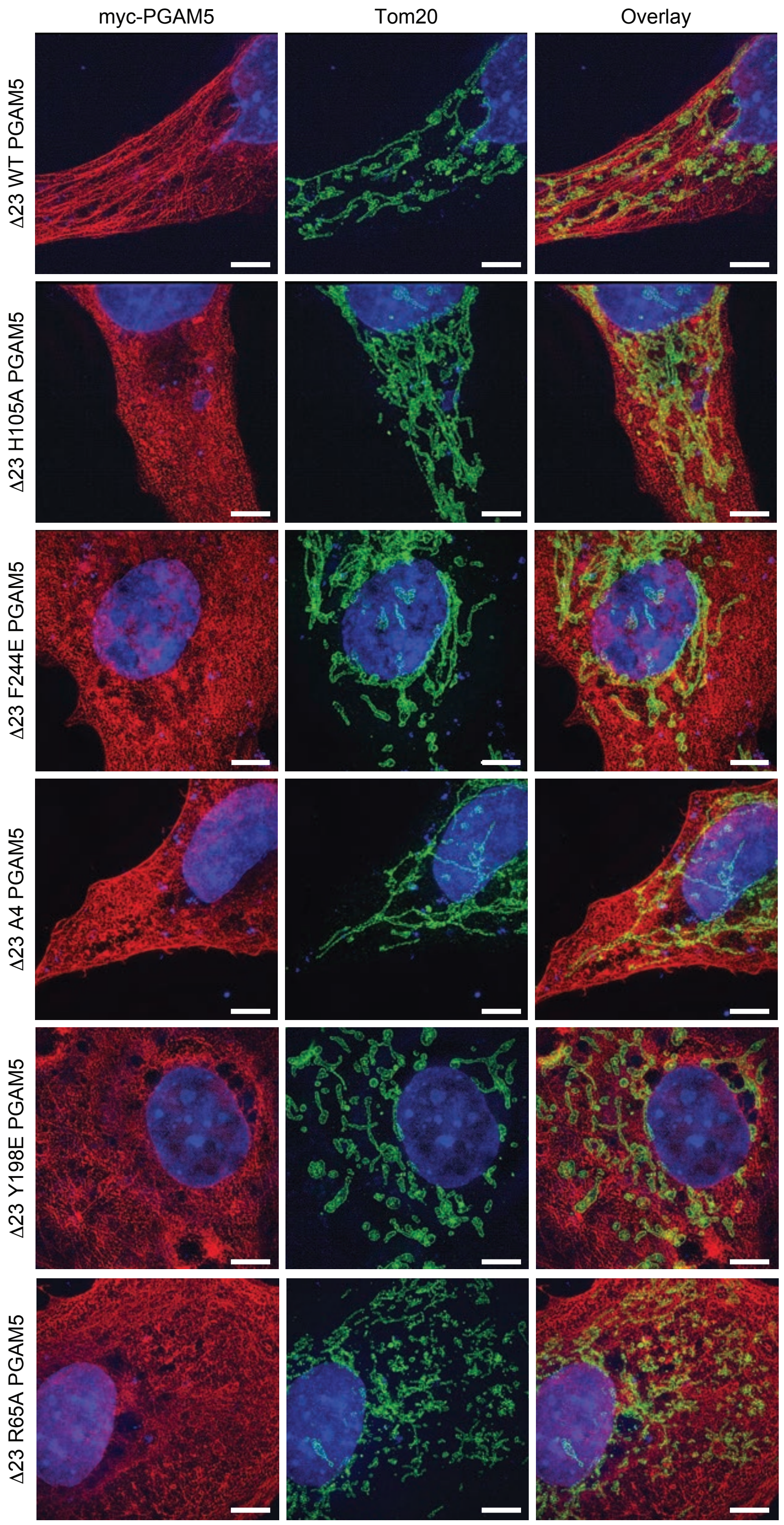

Supplementary Figure 7: Effect of dimerization interface mutations on PGAM5 filamentation. Representative structured illumination microscopy (SIM) images of COS7 cells transiently transfected with the myc-tagged Δ23 PGAM5 wild type (WT) and mutant variants. COS7 cells were immunostained for Tom20 with anti-Tom20 antibody (green), for PGAM5 with anti-myc antibody (red) and with DAPI (blue). All scale bars correspond to 10 μm.

Supplementary Figure 8

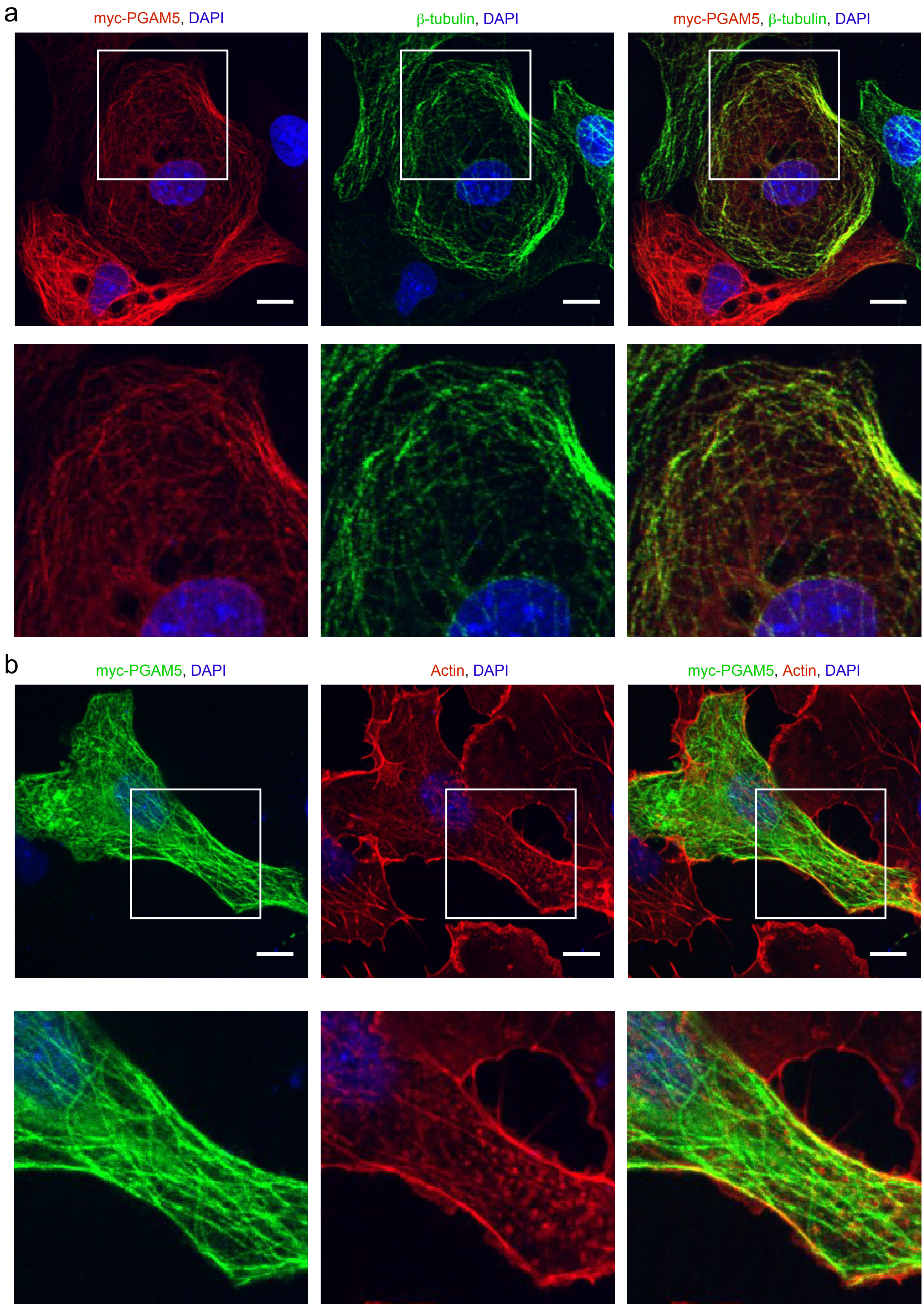

Supplementary Figure 8: Colocalization of PGAM5 filaments with cytoskeletal structures. a-b, Representative confocal images of COS7 cells transiently transfected with the myc-tagged Δ23 PGAM5 construct. **a,** COS7 cells were immunostained for microtubules with anti-β tubulin antibody (green), for PGAM5 with anti-myc antibody (red), and with DAPI (blue). **b,** COS7 cells were immunostained for actin with Alexa Fluor-647-phalloidin (red), for PGAM5 with anti-myc antibody (green) and with DAPI (blue). All scale bars correspond to 10 μm.

Supplementary Figure 9

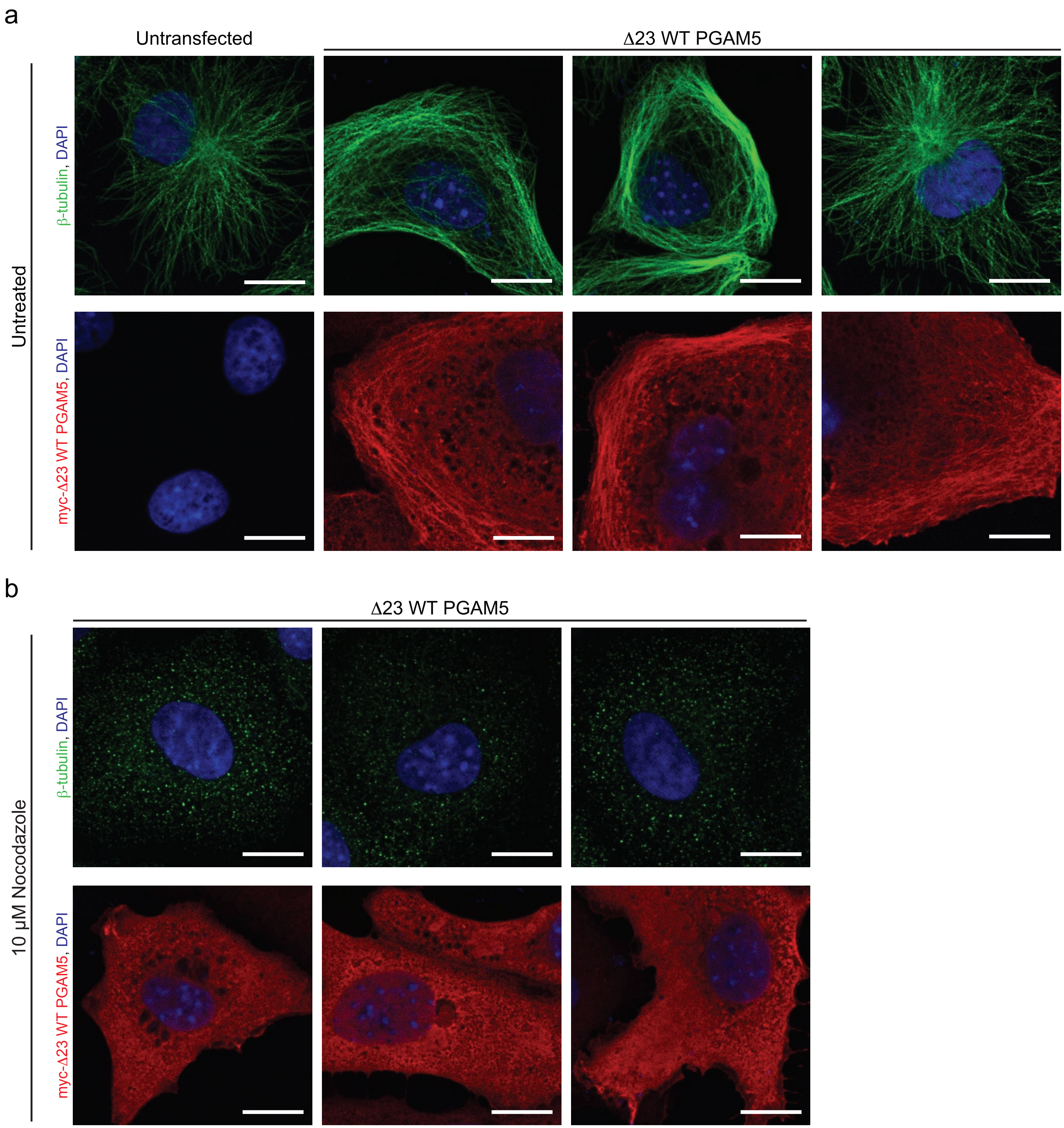

Supplementary Figure 9: Effect of nocodazole treatment on PGAM5 filaments. a-b, Representative confocal images of COS7 cells transiently transfected with myc-tagged Δ23 PGAM5. **a,** Untreated COS7 cells, either untransfected or transfected with the myc-tagged Δ23 PGAM5 wild type (WT). Upper panel: staining for β-tubulin with anti-β-tubulin antibody (green) and with DAPI (blue). Lower panel: staining for PGAM5 with anti-myc antibody (red) and with DAPI (blue). **b,** COS7 cells transfected with the myc-tagged Δ23 PGAM5 wild type (WT) treated with 10 μM Nocodazole for 45 minutes. Upper panel: staining for β-tubulin with anti-β-tubulin antibody (green) and with DAPI (blue). Lower panel shows a different set of cells stained for PGAM5 with anti-myc antibody (red) and with DAPI (blue). All scale bars correspond to 15 μm.

Supplementary Figure 10

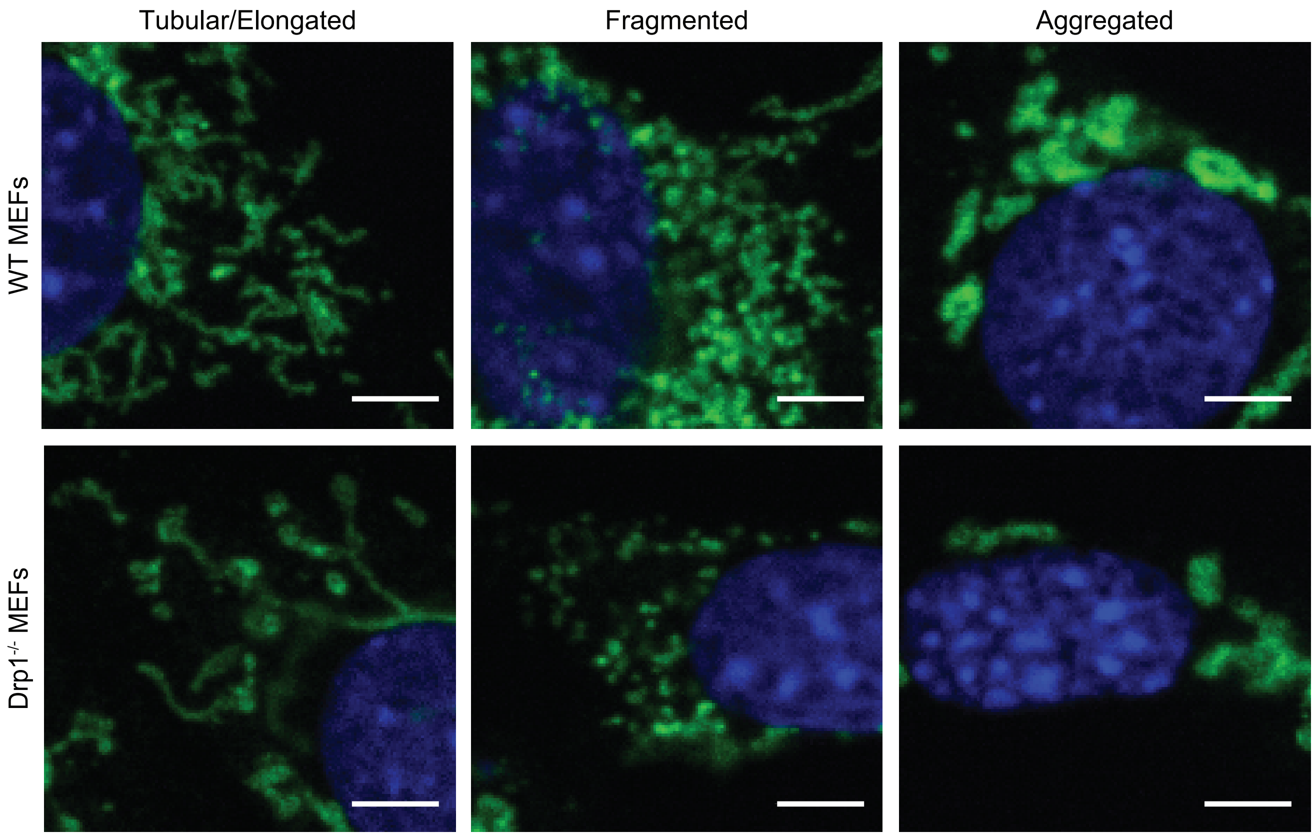

Supplementary Figure 10: Phenotypic categories used for scoring the effect of PGAM5 overexpression on mitochondrial morphology in MEF cells. Representative confocal images of the mitochondria in: untransfected wild type and Drp1$^{-/-}$ MEFs (left panels); wild type and Drp1$^{-/-}$ MEFs transiently transfected with full-length wild type PGAM5 (center panels) or wild type and Drp1$^{-/-}$ MEFs transiently transfected with full-length F244E PGAM5 (right panels). Cells were immunostained for Tom20 with anti-Tom20 antibody (green) and with DAPI (blue). All scale bars correspond to 15 μm.

Supplementary Figure 11

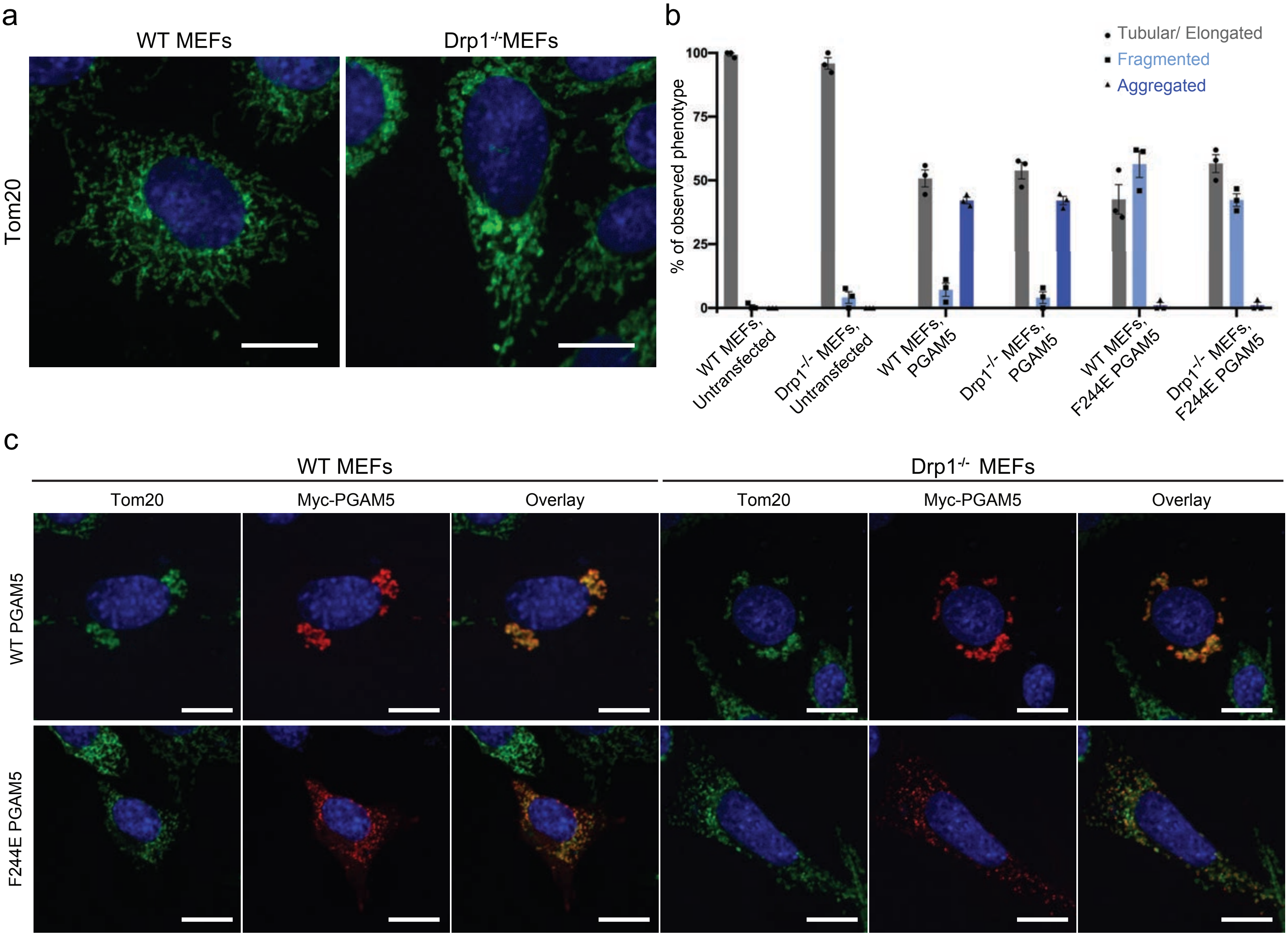

Supplementary Figure 11: DRP1 is dispensable for the clustered mitochondrial morphology induced by PGAM5.

a, Representative confocal images of wild type or Drp1$^{-/-}$ MEFs immunostained for the OMM marker Tom20 with anti-Tom20 antibody (green) and with DAPI (blue). Scale bars correspond to 15 µm. **b,** Quantification of mitochondrial phenotypes observed by confocal microscopy in wild type or Drp1$^{-/-}$ MEFs either untransfected or transiently transfected with the indicated PGAM5 constructs, based on at least 3 independent experiments per construct. Data are represented as mean +/- S.E.M, determined using GraphPad Prism. Untransfected wild type (WT) MEFs: n= 123 cells over 3 experimental replicates. WT MEFs expressing full-length WT PGAM5: n= 129 cells over 3 experimental replicates. WT MEFs expressing full-length F244E PGAM5: n= 89 cells over 3 experimental replicates. Untransfected Drp1$^{-/-}$ MEFs: n= 154 cells over 3 experimental replicates. Drp1$^{-/-}$ MEFs expressing full-length WT PGAM5: n= 1179 cells over 3 experimental replicates. Drp1$^{-/-}$ MEFs expressing F244E PGAM5: n= 89 cells over 3 experimental replicates. **c**, Representative confocal images of WT or Drp1$^{-/-}$ MEFs transiently transfected with the WT or dimer interface mutant (F244E) full-length myc-tagged PGAM5 and immunostained for the OMM marker Tom20 with anti-Tom20 antibody (green), for PGAM5 with anti-myc antibody (red) and with DAPI (blue). All scale bars correspond to 15 µm.

Supplementary Table 1. Cryo-EM data collection, refinement and validation statistics

	Δ48 PGAM5
**Data collection and processing**	
Magnification	22,500
Voltage (kV)	300
Electron exposure (e–/Å^2)	1.8
Defocus range (μm)	-1.0 – 2.5
Pixel size (Å)	0.53
Initial particle images (no.)	131,396
Final particle images (no.)	22,422
